# Sensitive and selective phenol sensing in denitrifying *Aromatoleum aromaticum* EbN1^T^

**DOI:** 10.1128/spectrum.02100-23

**Published:** 2023-10-12

**Authors:** Ramona Buschen, Pia Lambertus, Sabine Scheve, Simon Horst, Fei Song, Lars Wöhlbrand, John Neidhardt, Michael Winklhofer, Tristan Wagner, Ralf Rabus

**Affiliations:** 1 General and Molecular Microbiology, Institute for Chemistry and Biology of the Marine Environment (ICBM), Carl von Ossietzky University of Oldenburg, Oldenburg, Germany; 2 Human Genetics, Department of Human Medicine, Carl von Ossietzky University of Oldenburg, Oldenburg, Germany; 3 Research Center Neurosensory Science, Carl von Ossietzky University of Oldenburg, Oldenburg, Germany; 4 Sensory Biology of Animals, Institute of Biology and Environmental Sciences (IBU), Carl von Ossietzky University of Oldenburg, Oldenburg, Germany; 5 Max Planck Research Group Microbial Metabolism, Max Planck Institute for Marine Microbiology, Bremen, Germany; University of Minnesota Twin Cities, St. Paul, Minnesota, USA

**Keywords:** anaerobic degradation, phenol, alkylphenol, regulation, responsiveness, sensory system, 3D structure prediction, ligand binding, *Aromatoleum aromaticum *EbN1^T^

## Abstract

*Aromatoleum aromaticum* EbN1^T^ anaerobically degrades phenol, *p*-cresol, and *p*-ethylphenol, each via a distinct peripheral pathway. The compound-specific regulation of each pathway is proposed to occur on the transcriptional level in the case of phenol supposedly mediated by the one-component system PheR. To confirm its predicted function, we generated an unmarked, in-frame deletion mutant (Δ*pheR*). This mutant did not express the *ppsA1* gene, which encodes the A1 subunit of phenol-activating phenylphosphate synthase. The expression of *ppsA1* was restored by *in trans* complementation of *pheR* into the Δ*pheR* background. The responsiveness to phenol was studied *in vivo* in benzoate-limited anaerobic cultures by adding, upon benzoate depletion, single defined pulses of phenol (from 100 µM down to 0.1 nM). Time-resolved, targeted transcript profiling by qRT-PCR revealed a response threshold for *ppsA1* expression of 30‒50 nM phenol. Notably, *ppsA1* expression could not be induced by *p*-cresol or *p*-ethylphenol. Conversely, lack of expression was also observed for the additional target genes *cmh* (*p*-cresol degradation) and *acsA1* (*p*-ethylphenol degradation) applying phenol or *p*-ethylphenol as well as phenol or *p*-cresol as stimuli. Thus, the sensory proteins PheR, PcrS, and EtpR should be highly selective for phenol, *p*-cresol, and *p*-ethylphenol, respectively. The implicated incapability of cross-stimulus binding was corroborated by comparing the predicted 3D structural models of the proteins’ sensory domains. While the ligand-binding pockets share the conserved hydroxy group-anchoring histidine and tryptophane, their distal faces in PcrS and EtpR are, compared to PheR, enlarged to accommodate the bulkier methyl (*p*-cresol) and ethyl group (*p*-ethylphenol), respectively.

**IMPORTANCE:**

Aromatic compounds are globally abundant organic molecules with a multitude of natural and anthropogenic sources, underpinning the relevance of their biodegradation. *A. aromaticum* EbN1^T^ is a well-studied environmental betaproteobacterium specialized on the anaerobic degradation of aromatic compounds. The here studied responsiveness toward phenol in conjunction with the apparent high ligand selectivity (non-promiscuity) of its PheR sensor and those of the related *p*-cresol (PcrS) and *p*-ethylphenol (EtpR) sensors are in accord with the substrate-specificity and biochemical distinctiveness of the associated degradation pathways. Furthermore, the present findings advance our general understanding of the substrate-specific regulation of the strain’s remarkable degradation network and of the concentration thresholds below which phenolic compounds become essentially undetectable and as a consequence should escape substantial biodegradation. Furthermore, the findings may inspire biomimetic sensor designs for detecting and quantifying phenolic contaminants in wastewater or environments.

## INTRODUCTION

Phenolic compounds are widespread constituents of dissolved organic matter in the terrestrial and marine realm, with continuous input from natural as well as anthropogenic sources. They represent major building blocks of abundant biopolymers such as the plant cell wall inherent lignin (1) and are essential for sclerotizing the exoskeletons of insects (2). Phenolic compounds, as significant constituents of fossil energy sources, are released during extraction and processing [e.g., reference (3)]. Moreover, they are industrially produced in large quantities and represent bulk starting material for widely used phenolic resin-based plastics (4). Environmental and health concerns arise from their toxicity, carcinogenicity, and biologic resistance particularly in anoxic habitats (5), accounting for the need to fundamentally understand the biodegradation of these compounds, including the involved sensory/regulatory mechanisms.

The betaproteobacterial genus *Aromatoleum* encompasses a suite of denitrifying degradation specialists for recalcitrant aromatic, terpenoid, and hydrocarbon compounds (6). *Aromatoleum aromaticum* EbN1^T^ is particularly well-studied by proteogenomics with respect to the architecture of its complex, anaerobic degradation network, and the substrate-specific protein profiles of the network’s modules [e.g., references (7, 8)]. Furthermore, *A. aromaticum* EbN1^T^ stands out for its capacity to anaerobically degrade three structurally similar phenolic compounds (phenol, *p*-cresol, and *p*-ethylphenol) via distinct peripheral reaction sequences, which converge at 4-hydroxybenzoyl-CoA (Fig. S1). The latter is reductively dehydroxylated by the molybdenum cofactor-containing 4-hydroxybenzoyl-CoA reductase (HcrABC) (9) to the central intermediate benzoyl-CoA. The three peripheral degradation routes are as follows: (i) phenol is initially activated to phenylphosphate by ATP-dependent phenylphosphate synthase (PpsA1BC), followed by carboxylation to 4-hydroxybenzoate catalyzed by phenylphosphate carboxylase (PpcABCD). 4-Hydroxybenzoate is then activated to 4-hydroxybenzoyl-CoA by 4-hydroxybenzoate-CoA ligase (Hcl2). This pathway is analogous to the one originally elucidated in *Thauera aromatica* K172^T^ (10 – 12). (ii) *p*-Cresol is initially oxidized at its methyl group to the corresponding aldehyde by FAD-dependent *p*-cresol methylhydroxylase (Cmh) followed by dehydrogenation to 4-hydroxybenzoate catalyzed by 4-hydroxybenzaldehyde dehydrogenase (Hbd) and subsequent feeding into the phenol degradation pathway (7, 13). (iii) *p*-Ethylphenol is initially oxidized at its methylene group forming (*R*)-1-(4-hydroxyphenyl)ethanol by 4-ethylphenol methylenehydroxylase (EmhCF), followed by dehydrogenation to 4-hydroxyacetophenone catalyzed by (*R*)-specific 1-(4-hydroxyphenyl)ethanol dehydrogenase (Hped1). Then, consecutive carboxylation by 4-hydroxyacetophenone carboxylase (HacABC), activation to the respective CoA-ester by an acetoacetyl-CoA ligase-like protein (AcsA1), and final removal of acetyl-CoA by a predicted thiolase (TioL1) yield 4-hydroxybenzoyl-CoA (14 – 16).

**Fig 1:**
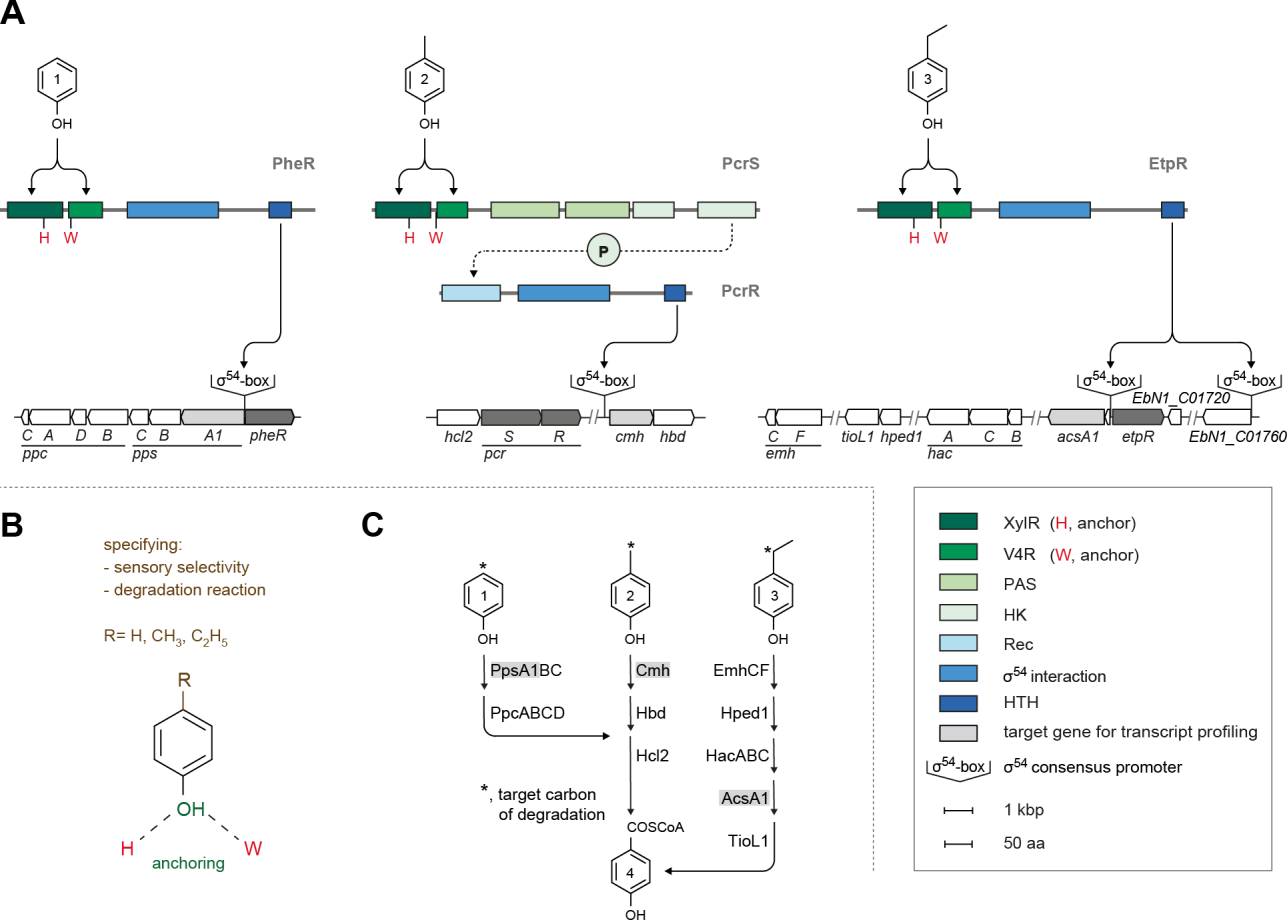
Sensory/regulatory circuits controlling anaerobic degradation of phenol, *p*-cresol, and *p*-ethylphenol in *Aromatoleum aromaticum* EbN1^T^. (**A**) Transcriptional control of the “catabolic” gene cluster for phenol is proposed to be mediated by the predicted one-component system PheR. In the case of *p*-cresol and *p*-ethylphenol, expression of their “catabolic” gene clusters is governed by the two-component system PcrSR and the one-component system EtpR, respectively (13, 22). Functional domains of PheR, PcrSR, and EtpR are color coded, and their coding genes are marked in dark gray. “Catabolic” genes selected for transcript profiling are highlighted in light gray. (**B**) Anchoring of the hydroxy group via conserved histidine and tryptophan residues in the sensory domain and *para* positioned moiety specifying the effector molecule. (**C**) Anaerobic degradation pathways of phenol, *p*-cresol, and *p*-ethylphenol; more detailed representation is provided in Fig. S1. Compound names: 1, phenol; 2, *p*-cresol; 3, *p*-ethylphenol; 4, 4-hydroxybenzoyl-CoA. Enzyme names: PpsA1BC, phenylphosphate synthase; PpcABCD, phenylphosphate carboxylase; Cmh, *p*-cresol methylhydroxylase; Hbd, 4-hydroxybenzaldehyde dehydrogenase; Hcl2, 4-hydroxybenzoate-CoA ligase; EmhCF, *p*-ethylphenol methylenehydroxylase; Hped1, 1-(4-hydroxyphenyl)ethanol dehydrogenase; HacABC, 4-hydroxyacetophenone carboxylase; AcsA1, acetoacetate-CoA ligase; TioL1, acyl-CoA C-acyltransferase. Domains: XylR, xylene regulator; V4R, 4-vinyl-reductase; PAS, Per-Arnt-Sim; HK, histidine kinase; Rec, receiver; HTH, helix-turn-helix.

Against the backdrop of the biochemical specificities of the aforementioned enzymes, their differential abundance and activity profiles (7, 14, 16), and the predicted sensory/regulatory proteins encoded in close proximity of the pathways’ genes (8, 17), it stands to reason that highly selective sensory recognition of phenol, *p*-cresol, and *p*-ethylphenol should govern a tight differential transcriptional control of the respective degradation pathways in *A. aromaticum* EbN1^T^ (Fig. 1). (i) The *pps-ppc* gene cluster for anaerobic phenol degradation harbors a σ^54^ consensus sequence in its promoter region and is directly flanked by the *pheR* gene, which encodes a predicted σ^54^-dependent one-component system (activator). Furthermore, the predicted PheR protein is homologous to the known phenol sensors MopR (from *Acinetobacter calcoaceticus*), CapR (also called DmpR, from *Pseudomonas putida*), and PoxR (from *Ralstonia eutropha*). Their determined crystal structures revealed conserved histidine and tryptophane residues in the N-terminal V4R domain, which anchor via hydrogen bridges the hydroxy group of phenol (18 – 21). (ii) The genes (*cmh*, *hbd*, and *hcl2*) assigned to anaerobic *p*-cresol degradation are intercalated by those for the predicted σ^54^-dependent two-component system PcrSR (activator), while the promotor region upstream of the *cmh* gene harbors a σ^54^ consensus promoter (13). (iii) The genes (*acsA1* through *emhCF*) for anaerobic *p*-ethylphenol degradation are preceded upstream by a σ^54^ consensus promoter and a gene encoding the σ^54^-dependent one-component system EtpR (repressor) (14). Notably, the sensory domains of PcrS and EtpR also contain a vinyl 4 reductase (V4R) domain with the conserved histidine and tryptophane residues, suggesting an analogous anchoring of their respective phenolic ligand. The predicted roles of PcrSR and EtpR as transcriptional regulators of their respective pathways were recently corroborated by in-frame deletions of their coding genes (13, 22). Moreover, the *in vivo* responsiveness of *A. aromaticum* EbN1^T^ toward *p*-cresol and *p*-ethylphenol (and its degradation intermediate *p*-hydroxyacetophenone), respectively, was recently shown to be in the lower nanomolar range (13, 23).

To advance the understanding of the regulation of the anaerobic degradation of phenolic compounds in *A. aromaticum* EbN1^T^, the present study pursued the following major aims: (i) substantiate the predicted role of PheR as transcriptional regulator of the “phenol-catabolic” genes by molecular genetic approaches; (ii) determine the *in vivo* responsiveness toward phenol; and (iii) investigate the sensory discrimination between phenol, *p*-cresol, and *p*-ethylphenol by differential expression profiling and comparing predicted 3D models of the respective sensory domains.

## RESULTS AND DISCUSSION

### Generation of ∆*pheR* and *pheR*-complemented mutants

**Fig 2:**
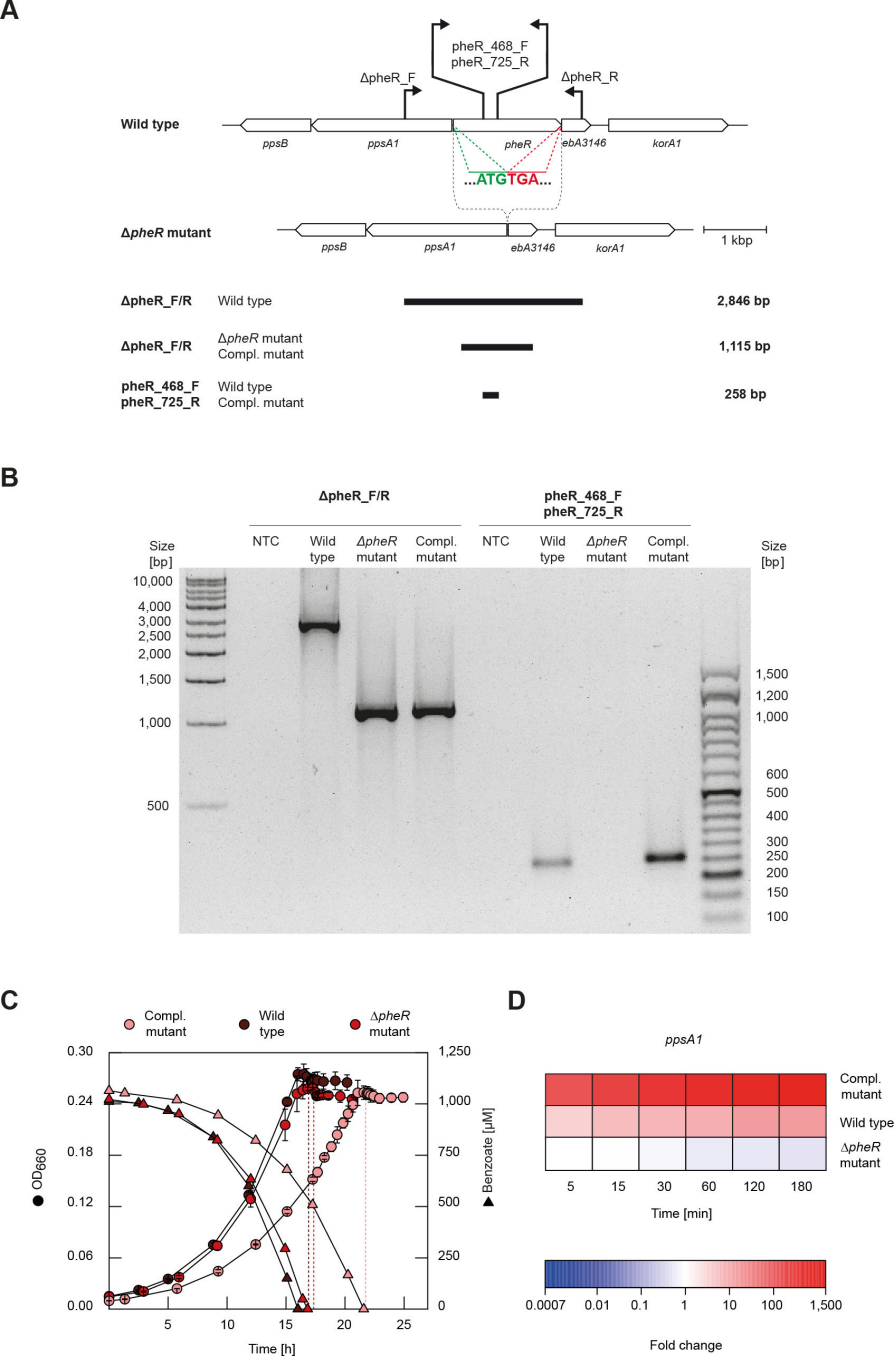
Generation of in-frame Δ*pheR* deletion and *pheR*-complemented (genotype: Δ*pheR*, pBBR1 MCS-2 ΩpheR) mutants of *A. aromaticum* EbN1^T^. (**A**) Scale model of the *pheR* gene and its 3′- and 5′-flanking regions on the chromosome, displaying the wild type (top) and the Δ*pheR* mutant (bottom). Chromosomal hybridization locations of the primer pairs ΔpheR_F/R and pheR_468_F/752_R to identify the deletion and complementation genotype are indicated by black arrows. Expected lengths of the respective PCR products are shown below the scale model. (**B**) Image of electrophoretically separated PCR products obtained from wild type, Δ*pheR* mutant, and *pheR*-complemented mutant. Applying the primer pair ΔpheR_F/R a 2,846 bp amplicon was obtained from the wild type, whereas the Δ*pheR* and complemented mutants both yielded 1,115 bp amplicons. Applying the primer pair pheR_468_F/752_R a 258-bp amplicon was obtained from wild type and *pheR*-complemented mutant, whereas no amplicon was obtained with the Δ*pheR* mutant. (**C**) Anaerobic growth of the Δ*pheR* and *pheR*-complemented mutants compared to the wild type under nitrate-reducing conditions with benzoate as sole source of carbon and energy. Upon benzoate depletion, a single pulse with phenol (100 µM) was given to each genotype (dashed lines). (**D**) Time-resolved transcript profile of the *ppsA1* gene in response to phenol, comparing wild type with the Δ*pheR* and *pheR*-complemented mutants. The *ppsA1* gene encodes the subunit A1 of phenylphosphate synthase, the initial enzyme of anaerobic degradation of phenol (see Fig. 1). qRT-PCR was used to determine relative transcript abundances, with the time point 5 min prior to phenol addition serving as reference. Determined fold changes rely each on three biological and three technical replicates and are provided in Table S1.

To verify the predicted role of the one-component system PheR in controlling the phenol-dependent expression of the “phenol-catabolic” gene cluster (Fig. 1A), a mutant with an unmarked in-frame Δ*pheR* deletion was generated. In the mutant, only the start and stop codons of the *pheR* gene were preserved in order to maintain the reading frame (Fig. 2A, marked in green and red, respectively). Accordingly, no amplicon could be observed using the primers specific for *pheR* in the deletion mutant, compared to the expected 258 bp product in the wild type. Using primers hybridizing up- and downstream of the deleted region, only a 1,115-bp amplicon was generated compared to a 2,846-bp product in the wild type (Fig. 2B). The newly generated Δ*pheR* mutant was *in trans*-complemented with the broad-host-range plasmid pBBR1 MCS-2 carrying the *pheR* gene. For the *pheR*-complemented mutant, a PCR product of 258 bp was obtained with gene-specific primers, which validates successful complementation with the *pheR* gene (Fig. 2B). The correctness of both mutants was verified by nucleotide sequencing.

Compared to the wild type, the Δ*pheR* mutant showed a similar growth behavior with benzoate and corresponding substrate consumption profile (Fig. 2C), indicating that the in-frame deletion does not affect the general growth physiology of *A. aromaticum* EbN1^T^. The *pheR*-complemented mutant attained the maximal optical density (OD_max_) ~5 h later than the other two genotypes, which is most likely due to the adverse effect of kanamycin added for selection reasons to the medium of this mutant.

### PheR mediates the expression of “phenol-catabolic” genes

The responsiveness to phenol of the Δ*pheR* and *pheR*-complemented mutants, compared to that of the wild type, was studied by the following two-stage experiment. First, non-adapted cells of *A. aromaticum* EbN1^T^ were anaerobically grown with a limited supply of benzoate (1 mM), upon the depletion of which (after ~17.2 h of incubation) a single pulse of 100 µM phenol was administered. Samples were retrieved 5 min prior to this pulse (reference) as well as 5, 15, 30, 60, 120, and 180 min after the pulse (test states). Second, for targeted transcript analysis by quantitative reverse transcription-PCR (qRT-PCR), the *ppsA1* gene located at the beginning of the “phenol-catabolic” gene cluster (Fig. 1A) was selected. Fold changes of transcript abundances are provided in Table S1 and illustrated in Fig. 2D. The lack of clear *ppsA1* expression in the Δ*pheR* mutant compared to the wild type upon the phenol pulse agrees with its predicted function as phenol-responsive transcriptional activator of the “phenol-catabolic” gene cluster. This is further corroborated by *ppsA1* expression exceeding the wild-type levels in the *pheR*-complemented mutant, which is probably due to the high-expression property of the involved *pheR*-carrying pBBR1 MCS-2 vector. Similar observations were previously reported in the context of mutant studies on *p*-cresol-sensing PcrSR (13) and *p*-ethylphenol-sensing EtpR (22).

### *In vivo* response threshold toward phenol

**Fig 3:**
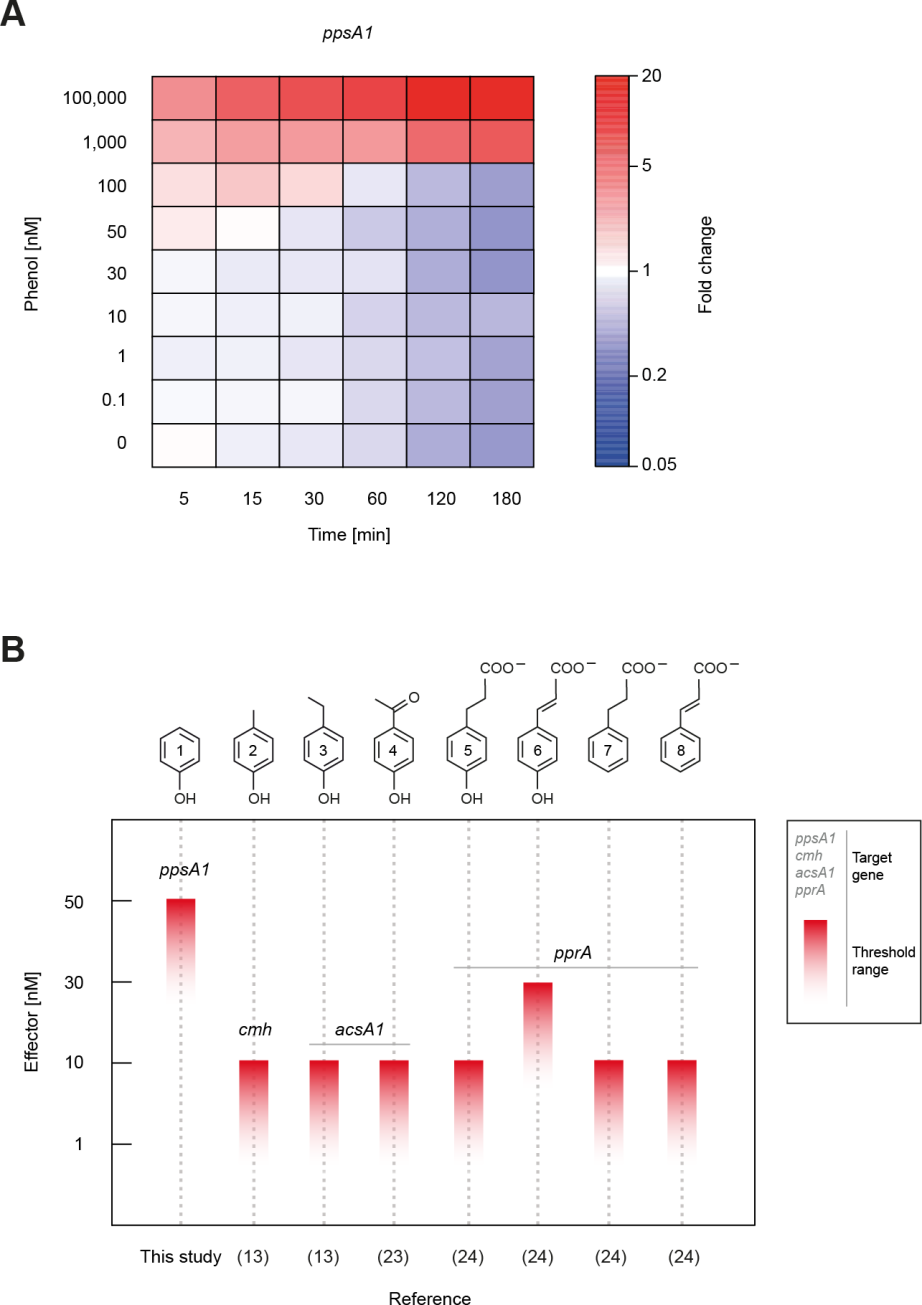
Transcriptional responsiveness of *A. aromaticum* EbN1 ^T^. (**A**) Time-resolved, quantitative transcript profiles of the *ppsA1* gene in response to different extracellular concentrations of phenol. The determination of relative transcript abundances was described in the legend to Fig. 2D, and fold changes are tabulated in Table S1. Each fold change relies on three biological and three technical replicates each. Growth cultures providing samples for the eight studied phenol concentrations as well as for the control (no addition of effector) are documented in Fig. S2. (**B**) Comparison of the response threshold determined here for phenol with those for other phenolic compounds and phenylpropanoids previously reported (13, 23, 24). Compound names: 1, phenol; 2, *p*-cresol; 3, *p*-ethylphenol; 4, 4-hydroxyacetophenone; 5, 3-(4-hydroxyphenyl)propanoate; 6, *p*-coumarate; 7, 3-phenylpropanoate; 8, cinnamate. Gene names: *ppsA1*, phenylphosphate synthase; *cmh*, *p*-cresol methylhydroxylase; *acsA1*, acetoacetate-CoA ligase*; pprA*, 3-phenylpropanoate-CoA ligase.

The response threshold of *A. aromaticum* EbN1^T^ wild type toward phenol was determined by applying the same two-stage experimental setup as described above for the mutant characterization. However, here, eight distinct phenol concentrations (from 100 µM down to 0.1 nM) were tested, for each of which an independent growth experiment with three replicate cultures was conducted (reproducibility documented in Fig. S2). Responsiveness was assessed by determining the expression profile of the *ppsA1* gene across the eight tested phenol concentrations and six test time points (Fig. 3A; Table S1). Constant levels of *ppsA1* transcripts at the reference time point (5 min prior to pulse) across all tested phenol concentrations afforded reliable comparison. A significant increase in *ppsA1* expression was observed already 5 min after 100 µM phenol was added, reaching its highest fold change (16-fold) after 120 min (Fig. 3A). The lowest pulse concentration still yielding a detectable transcriptional response was 50 nM, indicating the *in vivo* response threshold to range between 30 and 50 nM phenol. This threshold compares fairly well to those previously determined with *A. aromaticum* EbN1^T^ for other phenolic compounds (13) and various phenylpropanoids (24), which all fall in the lower nanomolar range (Fig. 3B).

### Physical considerations for cellular phenol uptake

Considering its uptake from the environment, phenol with its lipophilic character can be assumed to enter cells of *A. aromaticum* EbN1^T^ via passive diffusion across the cell envelope. Earlier theoretical considerations (13) suggest that equilibration between extra- and intracellular concentrations of alkylphenols occurs on millisecond time scales and is aided by their lipophilic nature, which enhances the effective permeability coefficient of the lipid bilayer membranes in the envelope by a factor of *K*_M_ (membrane partition coefficient). Compared to the two alkylated phenols *p*-cresol and *p*-ethylphenol studied earlier (13), phenol clearly has the lowest effective diffusion coefficient *D*_eff_ for passive diffusion across lipid bilayers (Table 1), which implies proportionately longer equilibration times, but still in the millisecond range. The experimentally determined threshold concentration for phenol is distinctly higher (30–50 nM) compared to *p*-cresol and *p*-ethylphenol (1–10 nM) (Fig. 3B), which we ascribe to lower binding affinity between phenol and its cognate sensor protein for the lack of other obvious reasons. Assuming that the threshold concentrations solely reflect differences in sensor-ligand-binding affinities, we can provide rough first estimates of the *K*_D_ values (Table 1), which also depend on the number of copies of a sensor protein in the cell before induction [for details, see reference (13)]. For essential proteins, a value of 10 copies per cell was found in *E. coli* (25), and we assume that of the 10 copies of a sensor protein present, at least 1 copy be bound to its ligand in order to have an effect on transcriptional regulation. This consideration leads to a *K*_D_ value in the submicromolar range.

**TABLE 1:** Physical considerations for cellular effector uptake^*a*^

Effector	*D* (10^−9^ m^2^ s^−1^)	*P*_ow_	*K*_M_	*D*_eff_ = *D* *K*_M_ (10^−9^ m^2^ s^−1^)	*c*_thresh_ (nM)	*N*_Sub_/cell	est. *K*_D_ (*N*_Sen_ = 10) (µM)
Phenol	1.0	32	7	7	30–50	50–83	0.3–0.5
*p*-Cresol	0.9	93	21	19	1–10	2–17	0.01–0.1
*p*-Ethylphenol	0.8 (est.)	320	74	59	1–10	2–17	0.01–0.1

^*a*^ D, Diffusion coefficient in water (26); *P*_ow_, octanol-water partition coefficient (27); *K*_M_, membrane partition coefficient, with *K*_M_ = 0.23 *P*_ow_ , see Eq. 8b in reference (13); *D*_eff_, effective diffusion coefficient in lipid membranes; *c*_thresh_, determined *in vivo* threshold concentration; *N*_Sub_, number of copies of substrate molecule per cell in equilibrium; *K*_D_, estimated upper bound for equilibrium dissociation constant, given observed threshold responses for transcriptional response, for *N*_Sen_ = 10 copies of sensor protein per cell.

### Negligible gratuitous induction by phenolic compounds

**Fig 4:**
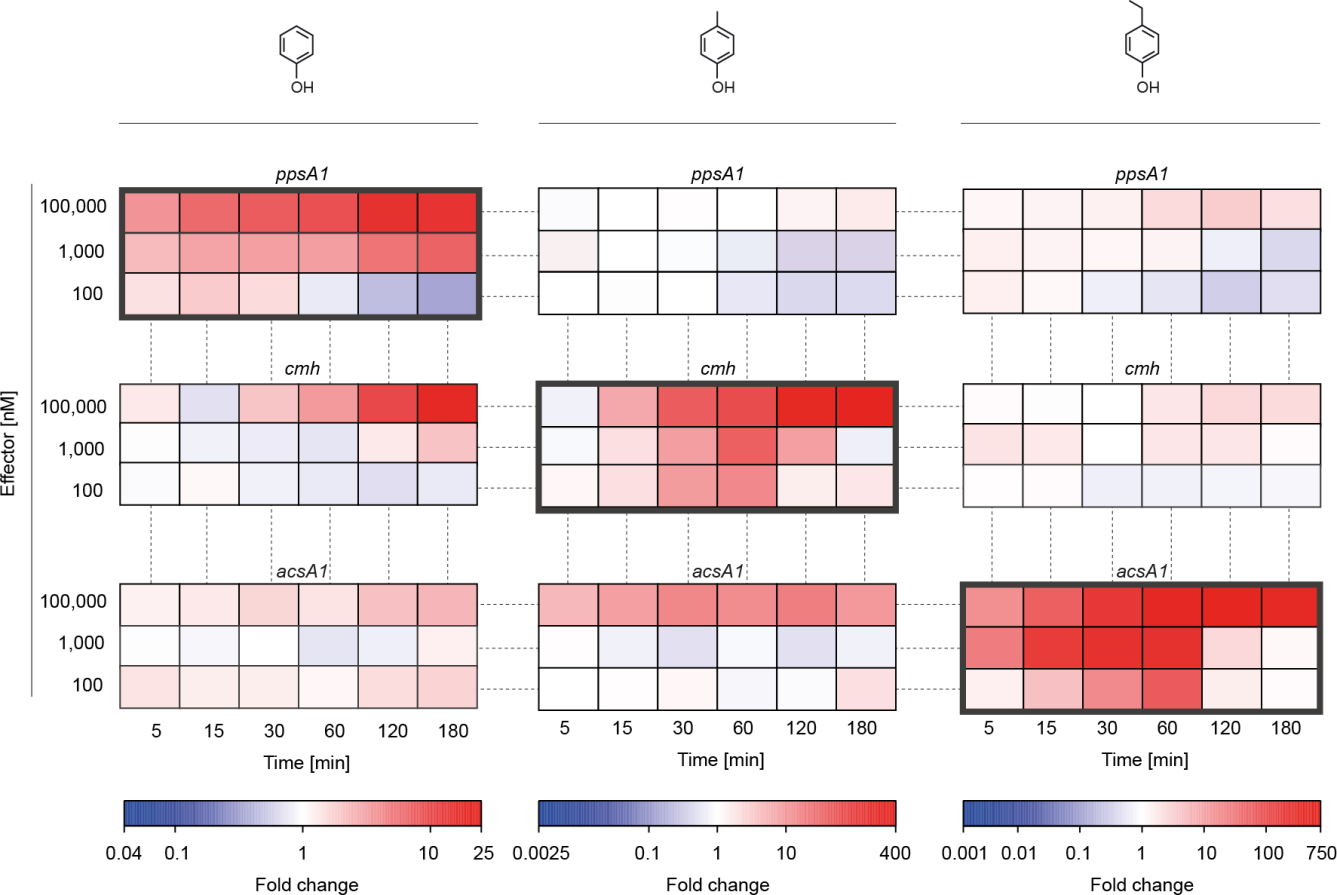
Negligible gratuitous induction by phenolic compounds in *A. aromaticum* EbN1^T^. Comparative, time-resolved, quantitative transcript profiles of the *ppsA1*, *cmh*, and *acsA1* genes in response to different concentrations of phenol, *p*-cresol, and *p*-ethylphenol. Gene names are as described in legend to Fig. 3. Determination of relative transcript abundances was as outlined in legend to Fig. 2D and fold changes are tabulated in Table S1. Each fold change relies on three biological and three technical replicates each. Note the differences in scale bars.

The chemical similarity of the three phenolic compounds supporting anaerobic growth of *A. aromaticum* EbN1^T^ prompts the study of their potential to cross-induce transcription of their non-associated “catabolic” gene clusters. This was tested by applying the same two-stage experimental setup as described above, but adding next to *pssA1* also *cmh* (for *p*-cresol) and *acsA1* (for *p*-ethylphenol) as target genes and next to phenol also *p*-cresol and *p*-ethylphenol as compounds to be tested. Since threshold determination was not relevant in this context, only three concentrations (100, 1, and 0.1 µM) were applied per compound. Overall, the transcript profiles delivered a uniform picture across the nine gene-vs-compound conditions (Fig. 4). Essentially, no expression of the *pssA1* gene could be detected when single pulses of *p*-cresol or *p*-ethylphenol were administered, except for a faint transcript formation at 100 µM *p*-ethylphenol recognizable only 60 min after the pulse. Expression of the *cmh* gene was clearly observed only ~60 min after the 100 µM phenol pulse and was merely close to background noise with *p*-ethylphenol. However, the essentially congruent *cmh* transcript abundance profiles at 100 µM phenol observed for Δ*pheR* mutant and wild type (Fig. S3) preclude a contribution of phenol-bound PheR to *cmh* expression and rather suggest a poor phenol-PcrS interaction only at higher ligand concentrations and after longer incubation time. Finally, low-level expression of the *acsA1* gene was only observed with 100 µM phenol or *p*-cresol. Considering the high-level expression of the target genes in response to the cognate phenolic compound, the aforementioned rare instances of cross-induced expression levels are ~1–2 orders of magnitude lower (Table S1). Taken together, a comparison of the transcript profiles suggests that the three tested phenolic compounds do not (or only very weakly) serve as gratuitous inducers for the non-cognate “catabolic” gene clusters. This could result from highly selective ligand-binding properties of the three involved sensory domains of the regulatory proteins (PheR, PcrS, and EtpR) as presented in the subsequent section “Predicted 3D structural models of sensory domains.”

To elaborate on the apparent lack of gratuitous induction by the three phenolic compounds, targeted transcript profiling was further expanded to the *bssA* and *ebdA1* genes of *A. aromaticum* EbN1^T^ (Fig. S4A). These encode the catalytic subunits of benzylsuccinate synthase and ethylbenzene dehydrogenase, which are key enzymes of the anaerobic degradation of toluene and ethylbenzene, respectively (Fig. S4B) (28, 29). Essentially, no significant expression of the *bssA* and *ebdA1* genes was observed, irrespective of the phenolic compound tested. This suggests that the involved sensory proteins TdiS (for toluene) and EdiS (for ethylbenzene), which are part of specific two-component systems (Fig. S5A), are not responsive to the phenolic compounds tested and that the regulatory domains of the PheR, PcrR, and EtpR proteins do not interact with the promoter regions of the “toluene- and ethylbenzene-catabolic” gene clusters.

### Predicted 3D structural models of sensory domains

To better understand the apparently high selectivity of the sensory domains of the PheR, PcrS, and EtpR proteins from *A. aromaticum* EbN1^T^, insights into their ligand-binding were aimed at. For this purpose, crystal structures of the sensory domains from the known phenol sensors MopR, CapR, and PoxR from other bacteria (18 – 21) were compared with 3D models of their counterparts in PheR, PcrS, and EtpR, which were generated by applying the recently available AlphaFold tool (30).

**Fig 5:**
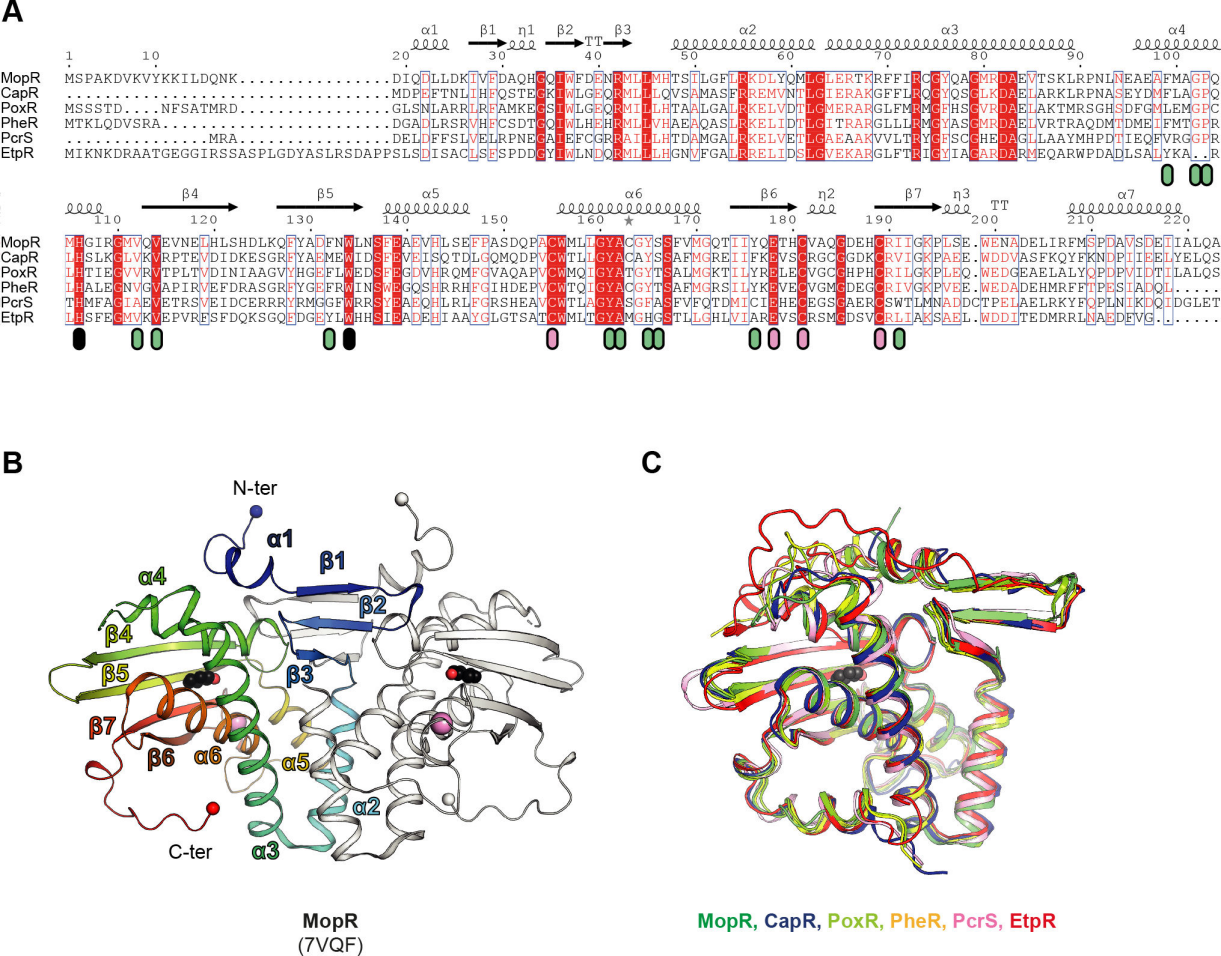
Similarities of the sensory domains from different sensory/regulatory proteins for phenolic compounds. (**A**) Sequence alignment superposed with the secondary structure elements derived from MopR (*Acinetobacter calcoaceticus*; PDB 7VQF) and generated by the Espript server. Symbols: red squares, perfect conservation; green and black ovals, van der Waals or hydrogen bond interaction with the phenol ligand; pink ovals, zinc ion-binding motif. Aligned proteins: MopR (PDB 7VQF) from *A. calcoaceticus*; CapR (PDB 6IY8) from *Pseudomonas putida*; PoxR (PDB 5FRW) from *Cupriavidus necator*; PheR (Q5P474), PcrS (Q5P0J1), and EtpR (Q5P8R7) from *A. aromaticum* EbN1^T^. (**B**) Overall fold of the MopR dimer in cartoon representation: one dimer is highlighted by a color gradient from blue (N-terminus) to red (C-terminus) and the other dimer is shown in gray. The nomenclature used for the secondary structure in panels A and B is from Ray et al. (21). Bound phenol is highlighted by black (carbon) and red (oxygen) spheres and the zinc atoms by pink spheres. (**C**) Overlay of the sensory domains of the six sensory/regulators proteins.

The multiple sequence alignment of the sensory domains of the six proteins (Fig. 5A; Fig. S5B) reveals, next to overall high similarities, several conserved residues that are functionally and structurally essential for the ligand-binding pocket: (i) a dyad His and Trp anchors the hydroxy group of phenol via hydrogen bonds, (ii) several hydrophobic residues that stabilize the aromatic ring of phenol via van der Waals interactions, and (iii) three Cys and one Glu residue(s) that are involved in tetrahedral zinc coordination for maintaining structural integrity. The overall fold of the sensory domains of the MopR dimer from *Acinetobacter calcoaceticus*, as revealed by its crystal structure (2.30 Å resolution) (19), is shown in Fig. 5B, highlighting bound phenol and zinc as well as indicating the secondary structural elements. The structural congruence of the sensory domains of MopR, CapR, PoxR, PheR, PcrS, and EtpR, in particular of the core area, is illustrated by an overlay image of the respective monomers in Fig. 5C. This forms a solid basis to reliably compare the ligand-binding pockets of the six sensory proteins in more detail.

**Fig 6:**
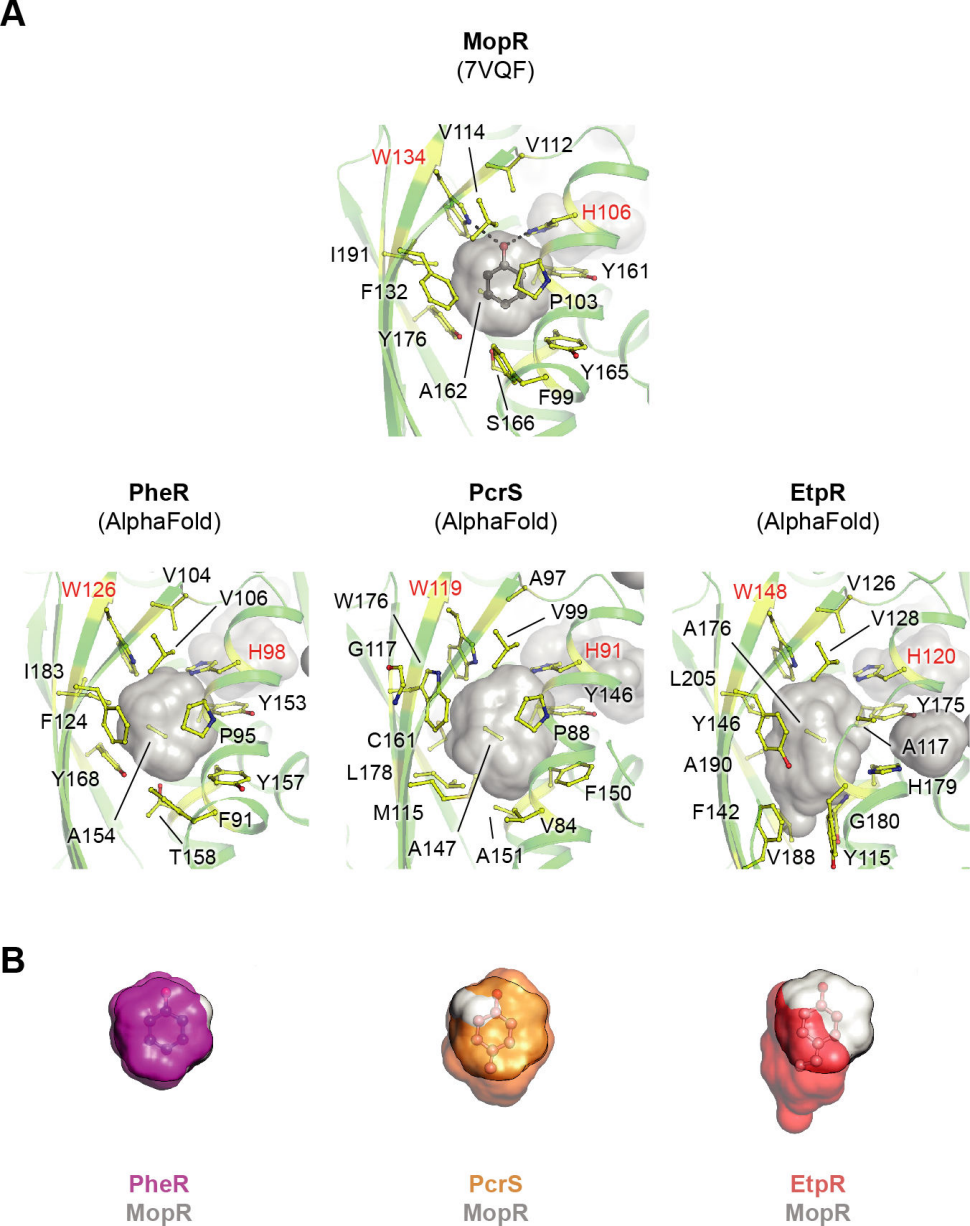
Shape and spatial expansion of binding pockets of phenolic compounds. (**A**) Zoom-ins into the binding pockets for phenolic compounds in the sensory domains of MopR [based on crystal structure from *A. calcoaceticus*; reference (19)] compared to the ones of PheR, PcrS, and EtpR from *A. aromaticum* EbN1^T^ based on AlphaFold predicted models. The modeled ligand-accommodating cavity is indicated by a transparent gray surface, while the residues enclosing the cavity are highlighted in balls and sticks. The conserved His and Trp residues anchoring the hydroxy group of the ligand are labeled in red, and the dashes show the hydrogen bonds in the experimental models. Phenol, modeled based on experimental data (MopR), has carbon colored in black and oxygen in red. (**B**) Comparison of the cavity volume between the experimental model MopR (gray surface) and the AlphaFold generated models of PheR, PcrS, and EtpR shown as transparent colored surfaces. The modeled phenol compounds are in transparent balls and sticks with carbon colored in black/pink and oxygen in red.

The experimentally determined structure of the phenol-binding pocket from MopR is enlarged in Fig. 6A (upper panel). Special emphasis is placed on the hydroxy group-anchoring Trp_134_ and His_106_ residues, the aromatic ring stabilizing hydrophobic residues (Phe_99_, Pro_103_, Val_112_, Val_114_, Phe_132_, Tyr_161_, Ala_162_, Tyr_165_, Tyr_176_, and Ile_191_), the Ser_166_, closing the pocket, as well as the shape of the ligand-accommodating cavity (transparent gray surface). The reliability of AlphaFold structures regarding the prediction of overall folds and binding pockets of the sensory domains becomes evident from their direct comparison with the respective crystal structures of the MopR, CapR, and PoxR dimers (Fig. S6 and S7). The structural congruency particularly applies to the decisive core area but is less pronounced for the functionally less relevant, more loosely structured N- and C-termini. The AlphaFold-predicted binding pockets of PheR, PcrS, and EtpR of *A. aromaticum* EbN1^T^ have an overall analogous shape to that of MopR, including the spatial positioning of the anchoring His and Trp residues (Fig. 6A, lower panel). While the ligand cavities of MopR and PheR are almost completely superimposable, in the case of PcrS and EtpR, differently sized spatial expansions are present at the opposite of the anchoring face (Fig. 6B). These expansions aptly accommodate the bulky methyl group of *p*-cresol (PheR) and ethyl group of *p*-ethylphenol (EtpR), while corroborating the observed non-cross induction by the three studied phenolic compounds in *A. aromaticum* EbN1^T^.

Furthermore, structural considerations also provide explanations for the apparent non-responsiveness of toluene-specific TdiS and ethylbenzene-specific EdiS sensory proteins toward the three tested phenolic compounds in *A. aromaticum* EbN1^T^. First, the V4R domain, which harbors the conserved, phenol-anchoring His and Trp residues, is absent from the sensory domains of TdiS and EdiS (Fig. S5A). Second, the dimeric structures of the sensory domains of phenol-sensing CapR [6IY8; reference (18)] and toluene-sensing PAS1 [5HWV; reference (31)], both from *Pseudomonas putida* and in the ligand-bound state, are highly dissimilar (Fig. S5C).

### Conclusions

*A. aromaticum* EbN1^T^ anaerobically degrades phenol, *p*-cresol, and *p*-ethylphenol via distinct peripheral reaction sequences, each comprising specific and, for the most part, biochemically intriguing enzymes. Therefore, it is economic for the cell to avoid the unproductive synthesis of such highly specialized enzymes if the native substrate is not available or present only at very low levels. *A. aromaticum* EbN1^T^ ensures this efficiency by means of highly substrate-specific, non-overlapping (non-promiscuous) regulatory circuits on the transcriptional level. The present study reveals that these circuits are controlled directly upfront by sensory/regulatory proteins (PheR, PcrSR, and EtpR), which are specifically tailored to recognize their cognate phenolic substrate based on its characteristic chemical feature.

The determined threshold of *A. aromaticum* EbN1^T^ for transcriptional responsiveness toward phenol under the applied cultivation conditions is in the nanomolar range (30‒50 nM) and somewhat higher than those previously reported for *p*-cresol and *p*-ethylphenol [1‒10 nM; reference (13)]. Such low thresholds possibly shed new light on the controlling factors for the persistence of dissolved organic matter as well as for the lower limits of bacterial growth with aromatic compounds. Furthermore, in synopsis with the here studied ligand-selectivity (non-promiscuity) of the involved sensory proteins, it appears unlikely that mixtures of phenolic growth substrates each available at nanomolar to lower micromolar concentrations could exert an amplifying effect on the sensory process.

## MATERIALS AND METHODS

### Bacterial strain and cultivation conditions

*A. aromaticum* EbN1^T^ (DSM 19018^T^) has been maintained in our laboratory since its isolation and was cultivated under nitrate-reducing conditions in defined, ascorbate-reduced, and bicarbonate-buffered mineral medium at 28°C as previously described (32). Aromatic growth substrates (benzoate, phenol, *p*-cresol, and *p*-ethylphenol) were added from aqueous stock solution sterilized by filtration. All cultivation experiments were started from glycerol stocks of the respective genotype of *A. aromaticum* EbN1^T^, following a multi-step procedure as recently described (13). Main cultures were performed in triplicates using 500 mL flat-bottomed glass bottles (400 mL culture volume) sealed with butyl rubber stoppers under an anoxic N_2_/CO_2_ (90:10, vol/vol) atmosphere, supplied with benzoate (1 mM) as sole source of carbon and energy, and inoculated with 2% (vol/vol) of active preculture. Cultures of the *pheR*-complemented mutant were supplemented with kanamycin (50 µg mL^‒1^) as selective marker. Growth was monitored by measuring the optical density of the cultures at 660 nm (OD_660_) (UVmini-1240; Shimadzu, Duisburg, Germany). Culture samples were retrieved, and substrate pulses were administered with sterile, N_2_-flushed syringes. All chemicals were of analytical grade.

### Generation of in-frame Δ*pheR* deletion mutation

Genomic DNA and plasmids were isolated according to standard protocols as previously described (33). For unmarked in-frame deletion of the *pheR* gene, a knockout plasmid was constructed on the basis of the suicide plasmid pK19mobsacB (34). The ﬁnal knockout plasmid contained 1.3 kbp of the upstream and 1.2 kbp of the downstream regions of the *pheR* gene. Both inserts were cloned simultaneously into pk19mobsacB using the In-Fusion HD cloning plus kit (TaKaRa Bio Inc., Kusatsu, Japan) according to the manufacturer’s instructions and using the PstI restriction site. For the cloning reaction, 100 ng of linearized pK19mobsacB and a molar ratio of 6:6:1 of the two inserts relative to the linearized plasmid were used. The respective primers were designed using the In-Fusion cloning primer design tool (TaKaRa Bio Inc.) and are given in Table 2. Homologous regions were amplified by PCR from genomic DNA of *A. aromaticum* EbN1^T^ using a high-fidelity polymerase (Phusion; Thermo Fisher Scientific, Dreieich, Germany). The ﬁnal knockout vector construct maintained the start and stop codons of the *pheR* gene. The knockout vector was transferred from *Escherichia coli* S17-1 to *A. aromaticum* EbN1^T^ via conjugation as described previously (33). Kanamycin-resistant single-crossover clones were veriﬁed by PCR. The single-crossover mutant was transferred into liquid medium (4 mM benzoate and 5 mM acetate) without kanamycin to induce the second crossover event, yielding either the Δ*pheR* or the wild-type genotype. Cells were plated on solid medium containing the same substrate mixture and 5% (wt/vol) sucrose. For identiﬁcation of the Δ*pheR* genotype, colonies were screened by PCR, yielding only an amplicon of 1,115 bp (Fig. 2AB).

**TABLE 2:** Oligonucleotide primers used for mutant construction

Primer^*a*^	Target gene^*b*^	Nucleotide sequence (3′→5′)	Product length (bp)
Generation of deletion mutant			
PheR(PstI) In1F	IR-*pheR*	TTACGCCAAGCTTGCATGCCTGCAGCACGGGTAGCGCGCCTTC	1,300
PheRIn1R	*pheR*	GCCGCGCTCACATGCCTCGCCCTCCTCC	
PheRIn2F	*pheR*	GCGAGGCATGTGAGCGCGGCAGCGGCGC	1,200
PheR(PstI) In2R	IR-*pheR*	GGGATCCTCTAGAGTCGACCTGCAGCCGGCTTTCGCGAGGTTCG	
Generation of complementation mutant			
PheRcompIn_Mcs2XbaI_F	*pheR*	TGGCGGCCGCTCTAGATTGTAATTCCGGTGTCGATCG	2,200
PheRcompIn_Mcs2XbaI_R		ATCCACTAGTTCTAGAGTGGTGCGTCTGCAGGTAC	

^*a*^ F, forward; R, reverse.

^*b*^ IR, intergenic region.

### Complementation of *pheR in trans* into the Δ*pheR* mutant

To achieve *in trans* expression of the *pheR* gene in the Δ*pheR* background, a complementation vector based on the broad-host-range vector pBBR1 MCS-2 conferring resistance to kanamycin (35) was generated. For this purpose, a 2.2-kbp fragment containing the *pheR* gene together with its ribosomal binding site was ampliﬁed from genomic DNA of *A. aromaticum* EbN1^T^ using a high-ﬁdelity polymerase (Phusion; Thermo Fisher Scientiﬁc) and the PheRcompIn_Mcs2XbaI_F/R primer pair, containing an XbaI restriction sites (Table 2). Purified PCR product and vector were digested with the restriction enzyme XbaI and purified. For ligation, digested PCR product and plasmid were mixed in a 4:1 ratio, denatured for 10 min at 65°C, and cooled on ice. Subsequently, T4-ligase (1 U) was added, mixed carefully, and incubated for 140 min at 24°C. The ligation reaction was transformed into chemical competent *E. coli* HST08. Single colonies grown on selective solid medium were screened and plasmids from positive clones were purified. The vector was transferred through a conjugation from the *E. coli* S17-1 background via agar-plate mating to the Δ*pheR* mutant, yielding the *pheR*-complemented mutant (genotype: Δ*pheR*, pBBR1 MCS-2 Ω*pheR*) and verified by PCR (Fig. 2B).

### Sequence validation of mutants by Sanger sequencing

For sequence validation of the Δ*pheR* mutant, a 2.5-kbp region of genomic DNA spanning across the entire up- and downstream regions, including the deletion site, was analyzed via Sanger sequencing as previously described (13). Additionally, a 2.7-kbp region of the complementation vector (Δ*pheR*, pBBR1 MCS-2 Ω*pheR*) spanning across the entire insert was sequenced to verify the *pheR*-complemented mutant. Genomic DNA was prepared according to standard methods (33). The fragments were ampliﬁed from genomic DNA and the complementation vector (pBBR1 MCS-2 Ω*pheR*) via PCR using a high-ﬁdelity polymerase (Phusion; Thermo Fisher Scientiﬁc) according to the manufacturer’s instructions and using the primer pairs provided in Table S2 (1F and 5R of each). For sequencing, samples were prepared using the BigDye buffer and BigDye terminator sequencing reagent and analyzed using a 3130xl genetic analyzer (Applied Biosystems, Waltham, MA, USA) as described by the manufacturer.

### Growth experiments for mutant characterization

To assess the effect of the *pheR* deletion, the three genotypes (wild type, Δ*pheR* mutant, and *pheR*-complemented mutant) of *A. aromaticum* EbN1^T^ were grown with benzoate (1 mM). Replicate cultures were sampled in short intervals to monitor growth (OD_660_) and substrate consumption. After complete depletion of benzoate, a pulse of 100 µM phenol was given. For each experiment and replicate, a sample taken 5 min prior to the pulse served as reference state, while samples retrieved 5, 15, 30, 60, 120, and 180 min after the pulse represented the test states. Five milliliters of samples was immediately added to 10 mL of RNAprotect bacterial reagent (Qiagen, Hilden, Germany), mixed rigorously, incubated for 5 min at room temperature, and then centrifuged (4,000 × *g*, 10 min, 4°C). Pellets were resuspended in 0.5 mL RNAprotect bacterial reagent, transferred into 2 mL microcentrifuge tubes, and centrifuged (20,000 × *g*, 10 min, room temperature). Supernatants were discarded, and pellets were shock frozen in liquid N_2_ and stored at ‒80°C until further analyses.

### Growth experiments for determining phenol response threshold

The responsiveness of *A. aromaticum* EbN1^T^ to various concentrations of phenol was studied with non-adapted cells. Cultivation was performed as described above, with three replicate cultures performed per tested phenol concentration. Upon depletion of the primary growth substrate benzoate (highly reproducible after ~17.2 h of incubation; Fig. S2), a single pulse of phenol at a defined concentration was administered (100 µM, 1 µM, 100 nM, 50 nM, 30 nM, 10 nM, 1 nM, or 0.1 nM). A negative-control experiment without the addition of phenol was performed under the same conditions. Throughout the incubation time, 3 mL samples of the culture broth were retrieved to monitor growth: a 1 mL aliquot for measuring OD_660_ and a 2 mL aliquot for determining benzoate depletion. In the case of the latter, the samples were immediately centrifuged (20,000 × *g*, 10 min, 4°C) and the supernatants were stored at ‒20°C for subsequent analyses by micro-high-performance liquid chromatography (microHPLC). For transcript profiling, additional 5 mL samples was retrieved at each of the seven time points detailed in above paragraph on “Growth experiments for mutant characterization.” Sample treatment and storage was likewise performed.

### Growth experiments for assessing sensory selectivity

To determine the transcriptional responsiveness of the *ppsA1*, *cmh, acsA1*, *bssA*, and *ebdA1* genes to phenol, *p*-cresol, and *p*-ethylphenol, essentially, the same experimental setup was used as described in the two paragraphs above (Fig. 4). Here, however, for each of the separately administered phenolic compounds, only three different concentrations were applied (100 µM, 1 µM, and 100 nM).

### Quantiﬁcation of benzoate by (micro)HPLC

Quantitative depletion proﬁling of benzoate was achieved using a microHPLC system (UltiMate 3000; Thermo Fisher Scientiﬁc, Germering, Bavaria, Germany) as previously described (23). The system was equipped with a reverse-phase C_18_ column (Thermo Hypersil Gold; Thermo Fisher Scientific) and a diode array detector (DAD-3000; Thermo Fisher Scientific) and operated at 40°C with a ﬂow rate of 0.1 mL min^‒1^. Eluent A was composed of 5% (vol/vol) acetonitrile in H_2_O with 0.01% (vol/vol) H_3_PO_4_ (85%) and eluent B of 90% (vol/vol) acetonitrile in H_2_O with 0.01% (vol/vol) H_3_PO_4_ (85%). The 20 min gradient was as follows: 2.5 min constant at 3% B, 4 min linear ramping to 65% B, 1 min linear ramping to 99% B, 1.5 min constant at 99% B, 2 min linear ramping to 3% B, and 9 min constant at 3% B. Benzoate was detected at 229 nm, with a retention time of 9.67 min and a dynamic range from 5 nM to 50 µM.

### Preparation of total RNA

Preparation of total RNA was performed according to the protocol of Oelmüller et al. (36) and as previously described (13), using cells treated with RNAprotect Bacteria Reagent and stored at ‒80°C. A given cell pellet was resuspended in STE buffer (10 mM Tris-HCl, 1 mM EDTA, 100 mM NaCl, pH 8.3). Then, 20 µL SDS (10%, wt/vol) and 900 µL Roti Aqua-Phenol (CarlRoth, Karlsruhe, Germany) were added; the suspension was incubated at 60°C for 8 min by gently inverting and then centrifuged (20,000 × *g*, 5 min, room temperature). The resultant aqueous phase was transferred into a 2-mL 5PRIME phase lock gel tube (Quantabio, Beverly, MA, USA). One volume of phenol-chloroform-isoamylalcohol (25:24:1) was added, and the tube was gently inverted for 5 min. After centrifugation (20,000 × *g*, 5 min, room temperature), nucleic acids were precipitated using ice-cold ethanol (96%) during incubation at −80°C for 30 min. After centrifugation (20,000 × *g*, 30 min, 4°C), the pellet was washed with ice-cold ethanol (75%) and centrifuged again (20,000 × *g*, 15 min, 4°C). The pellet was dried and suspended in RNase-free water. Subsequently, the sample was digested with DNase I (RNase-free; Qiagen). Complete removal of DNA was conﬁrmed by PCR using genomic DNA of *A. aromaticum* EbN1^T^ as a positive control. RNA quality underlying the transcript profiles was controlled using an Experion automated electrophoresis station (Bio-Rad, Hercules, CA, USA), confirming the integrity of rRNAs as well as the ratio between 23S and 16S rRNA. RNA concentrations were determined using a microplate reader (SPECTROstar Nano; BMG Labtech, Ortenberg, Germany). RNA samples were stored at −80°C until further analyses. All chemicals used for RNA preparation were of molecular biology grade.

### Transcript proﬁling by qRT-PCR

Relative expression levels of different target genes in the three genotypes were determined by real-time RT-PCR. For the selected target genes, speciﬁc primers (Table 3) were designed using the software Primer3 (version 4.1.0; https://primer3.ut.ee/). cDNA generation and real-time PCR were performed with three technical replicates per RNA preparation applying 150 ng of total RNA and using the Brilliant III ultra-fast SYBR green quantitative reverse transcription-PCR (qRT-PCR) master mix (Agilent, Santa Clara, CA, USA) and the CFX96 real-time system (Bio-Rad). In total, nine measurements were conducted per analyzed time point. The one-tube RT real-time PCR was carried out with one cycle of reverse transcription for 10 min at 50°C and one cycle of PCR initiation for 3 min at 95°C, followed by 40 cycles of 10 s of denaturation at 95°C, 30 s of annealing (primer speciﬁc), and 30 s of extension at 60°C, succeeded by real-time detection for 5 s. The gene-speciﬁc annealing temperatures were the following: 60°C for *acsA1, cmh, ebdA1*, and *ppsA1*; 64°C for *bssA*. The speciﬁcity of accumulated products was veriﬁed by melting curve analysis, ranging from 60°C to 90°C in steps of 0.5°C. Differences in relative transcript abundance between reference and test states were calculated as previously described (13) according to the following equation: Ratios = *E* ^Δ*CT*(reference - test)^. Primer-specific efficiencies (*E*) (Table 3) were determined as described previously (13, 37).

**TABLE 3:** Oligonucleotide primers used for targeted transcript profiling

Primer^*a*^	Target gene	Nucleotide sequence (3′→5′)	Product length (bp)	Primer-specific efficiency (*E*)
PpsA_F PpsA_R	*ppsA1*	TCTGGTTCTACGACGGACTG CAGGTGATAGCCCTTCGACT	391	1.92
Cmh_991_F Cmh_1241_R	*cmh*	GAAACCAACGACGCCAAC ATCGTCTTCGCCATCTGC	251	1.97
Acsa1_87F Acsa1_336R	*acsA1*	AGACACCCGTAAGCTGAAATTTG GTTCTCGCTCAGATACATGATGG	249	1.96
EbdA_2082_F EbdA_2590_R	*ebdA1*	TCTCAAGAAGGTCGGGGAAC GATGGGAATTCGTGAGGTGC	508	1.95
Bssa_1658_F Bssa_1952_R	*bssA*	CGTTCCGCAAGCAGTACC TAGCCTTCCCAGTTCGCC	294	1.88

^*a*^ F, forward; R, reverse.

### Prediction of 3D structure models

The crystal structures of the sensory domains of MopR (7VQF and 5KBE), CapR (6IY8), and PoxR (5FRW) were obtained from the RCSB protein database (URL: RCSB.org) (38). The structural models were predicted using the neural network-based model AlphaFold (30) and its related database (39) by applying the prediction based on a homodimer generation. Graphical representation of overall folds and zoom-ins of the binding pockets were generated using Pymol (the PyMOL Molecular Graphics System, version 2.2.0; Schrödinger, New York, NY, USA). Multiple sequence alignment of the sensory domains from selected proteins was generated by ESPript (https://espript.ibcp.fr) (40).

## Data Availability

All data this study builds on are presented in the manuscript and supplemental material.

## SUPPLEMENTAL MATERIAL

Fig. S1 spectrum.02100-23-s0001.pdf Anaerobic degradation pathways for phenol, *p*-cresol, and *p*-ethylphenol.

Fig. S2 spectrum.02100-23-s0002.pdf Anaerobic cultivation of *A. aromaticum* EbN1^T^ for targeted transcript analysis in response to phenol.

Fig. S3 spectrum.02100-23-s0003.pdf Transcript profiles of the *cmh* gene in wild type and Δ*pheR* mutant of *A. aromaticum* EbN1^T^ after a pulse with 100 *μ*M phenol.

Fig. S4 spectrum.02100-23-s0004.pdf No gratuitous induction of toluene- and ethylbenzene-catabolic genes with phenolic compounds in *A. aromaticum* EbN1^T^.

Fig. S5 spectrum.02100-23-s0005.pdf Rationale for incapacity of toluene and ethylbenzene sensory proteins to respond to phenolic compounds in *A. aromaticum* EbN1^T^.

Fig. S6 spectrum.02100-23-s0006.pdf Shape and spatial expansion of phenol-binding pockets comparing models from crystal structures and AlphaFold predictions.

Fig. S7 spectrum.02100-23-s0007.pdf Similarities of the overall sensory domain structures from different sensory/regulatory proteins for phenolic compounds comparing models from crystal structures and AlphaFold predictions.

Table S1 spectrum.02100-23-s0008.xlsx Transcript profiles of *ppsA1*, *cmh*, *acsA1*, *ebdA1*, and *bssA* genes upon pulses with phenol, *p*-cresol, or *p*-ethylphenol.

Table S2 spectrum.02100-23-s0009.xlsx Oligonucleotide primers used for nucleotide sequencing.

ASM does not own the copyrights to Supplemental Material that may be linked to, or accessed through, an article. The authors have granted ASM a non-exclusive, world-wide license to publish the Supplemental Material files. Please contact the corresponding author directly for reuse.

## References

[1] Lin D , Jiang S , Zhang A , Wu T , Qian Y , Shao Q . 2022. Structural derivatization strategies of natural phenols by semi-synthesis and total-synthesis. Nat Prod Bioprospect 12:8. doi:10.1007/s13659-022-00331-6 35254538 PMC8901917

[2] Peter MG . 1993. Die molekulare Architektur des Exoskeletts von Insekten. Chem Unserer Zeit 27:189–197. doi:10.1002/ciuz.19930270404

[3] Giabbai MF , Cross WH , Chian ESK , Dewalle FB . 1985. Characterization of major and minor organic pollutants in wastewater from coal gasification processes. Int J Environ Anal Chem 20:113–129. doi:10.1080/03067318508077050

[4] Pilato L . 2010. Phenolic resins: a century of progress. Springer, Berlin, Heidelberg. doi:10.1007/978-3-642-04714-5

[5] ATSDR. 2008. Toxicological profile for phenol. US Department of Health and Human Services.

[6] Rabus R , Wöhlbrand L , Thies D , Meyer M , Reinhold-Hurek B , Kämpfer P . 2019. Aromatoleum gen. nov., a novel genus accommodating the phylogenetic lineage including Azoarcus evansii and related species, and proposal of Aromatoleum aromaticum sp. nov., Aromatoleum petrolei sp. nov., Aromatoleum bremense sp. nov., Aromatoleum toluolicum sp. nov. and Aromatoleum diolicum sp. nov. Int J Syst Evol Microbiol 69:982–997. doi:10.1099/ijsem.0.003244 30762514

[7] Wöhlbrand L , Kallerhoff B , Lange D , Hufnagel P , Thiermann J , Reinhardt R , Rabus R . 2007. Functional proteomic view of metabolic regulation in "Aromatoleum aromaticum" strain EbN1. Proteomics 7:2222–2239. doi:10.1002/pmic.200600987 17549795

[8] Rabus R , Trautwein K , Wöhlbrand L . 2014. Towards habitat-oriented systems biology of "Aromatoleum aromaticum" EbN1. Chemical sensing, catabolic network modulation and growth control in anaerobic aromatic compound degradation. Appl Microbiol Biotechnol 98:3371–3388. doi:10.1007/s00253-013-5466-9 24493567

[9] Boll M , Fuchs G , Meier C , Trautwein A , El Kasmi A , Ragsdale SW , Buchanan G , Lowe DJ . 2001. Redox centers of 4-hydroxybenzoyl-CoA reductase, a member of the xanthine oxidase family of molybdenum-containing enzymes. J Biol Chem 276:47853–47862. doi:10.1074/jbc.M106766200 11602591

[10] Biegert T , Altenschmidt U , Eckerskorn C , Fuchs G . 1993. Enzymes of anaerobic metabolism of phenolic compounds. 4-Hydroxybenzoate-CoA ligase from a denitrifying Pseudomonas species. Eur J Biochem 213:555–561. doi:10.1111/j.1432-1033.1993.tb17794.x 8477728

[11] Schmeling S , Narmandakh A , Schmitt O , Gad’on N , Schühle K , Fuchs G . 2004. Phenylphosphate synthase: a new phosphotransferase catalyzing the first step in anaerobic phenol metabolism in Thauera aromatica. J Bacteriol 186:8044–8057. doi:10.1128/JB.186.23.8044-8057.2004 15547277 PMC529068

[12] Schühle K , Fuchs G . 2004. Phenylphosphate carboxylase: a new C-C Lyase involved in anaerobic phenol metabolism in Thauera aromatica. J Bacteriol 186:4556–4567. doi:10.1128/JB.186.14.4556-4567.2004 15231788 PMC438602

[13] Vagts J , Weiten A , Scheve S , Kalvelage K , Swirski S , Wöhlbrand L , Neidhardt J , Winklhofer M , Rabus R . 2020. Nanomolar responsiveness of an anaerobic degradation specialist to alkylphenol pollutants. J Bacteriol 202:e00595-19. doi:10.1128/JB.00595-19 31843798 PMC7015708

[14] Wöhlbrand L , Wilkes H , Halder T , Rabus R . 2008. Anaerobic degradation of p-ethylphenol by "Aromatoleum aromaticum" strain EbN1: pathway, regulation, and involved proteins. J Bacteriol 190:5699–5709. doi:10.1128/JB.00409-08 18539747 PMC2519382

[15] Büsing I , Höffken HW , Breuer M , Wöhlbrand L , Hauer B , Rabus R . 2015. Molecular genetic and crystal structural analysis of 1-(4-hydroxyphenyl)-ethanol dehydrogenase from 'Aromatoleum aromaticum' EbN1. J Mol Microbiol Biotechnol 25:327–339. doi:10.1159/000439113 26488297

[16] Muhr E , Schühle K , Clermont L , Sünwoldt K , Kleinsorge D , Seyhan D , Kahnt J , Schall I , Cordero PR , Schmitt G , Heider J . 2015. Enzymes of anaerobic ethylbenzene and p-ethylphenol catabolism in 'Aromatoleum aromaticum': differentiation and differential induction. Arch Microbiol 197:1051–1062. doi:10.1007/s00203-015-1142-z 26275558

[17] Rabus R , Kube M , Heider J , Beck A , Heitmann K , Widdel F , Reinhardt R . 2005. The genome sequence of an anaerobic aromatic-degrading denitrifying bacterium, strain EbN1. Arch Microbiol 183:27–36. doi:10.1007/s00203-004-0742-9 15551059

[18] Park K-H , Kim S , Lee S-J , Cho J-E , Patil VV , Dumbrepatil AB , Song H-N , Ahn W-C , Joo C , Lee S-G , Shingler V , Woo E-J . 2020. Tetrameric architecture of an active phenol-bound form of the AAA+ transcriptional regulator DmpR. Nat Commun 11:2728. doi:10.1038/s41467-020-16562-5 32483114 PMC7264223

[19] Singh J , Sahil M , Ray S , Dcosta C , Panjikar S , Krishnamoorthy G , Mondal J , Anand R . 2022. Phenol sensing in nature is modulated via a conformational switch governed by dynamic allostery. J Biol Chem 298:102399. doi:10.1016/j.jbc.2022.102399 35988639 PMC9556785

[20] Patil VV , Park K-H , Lee S-G , Woo E . 2016. Structural analysis of the phenol-responsive sensory domain of the transcription activator PoxR. Structure 24:624–630. doi:10.1016/j.str.2016.03.006 27050690

[21] Ray S , Gunzburg MJ , Wilce M , Panjikar S , Anand R . 2016. Structural basis of selective aromatic pollutant sensing by the effector binding domain of MopR, an NtrC family transcriptional regulator. ACS Chem Biol 11:2357–2365. doi:10.1021/acschembio.6b00020 27362503

[22] Büsing I , Kant M , Dörries M , Wöhlbrand L , Rabus R . 2015. The predicted σ5^4-^dependent regulator EtpR is essential for expression of genes for anaerobic p-ethylphenol and p-hydroxyacetophenone degradation in "Aromatoleum aromaticum" EbN1. BMC Microbiol 15:251. doi:10.1186/s12866-015-0571-9 26526497 PMC4630880

[23] Vagts J , Scheve S , Kant M , Wöhlbrand L , Rabus R . 2018. Towards the response threshold for p-hydroxyacetophenone in the denitrifying bacterium "Aromatoleum aromaticum" EbN1. Appl Environ Microbiol 84:e01018-18. doi:10.1128/AEM.01018-18 29959253 PMC6121972

[24] Vagts J , Kalvelage K , Weiten A , Buschen R , Gutsch J , Scheve S , Wöhlbrand L , Diener S , Wilkes H , Winklhofer M , Rabus R . 2021. Responsiveness of Aromatoleum aromaticum EbN1^T^ to lignin-derived phenylpropanoids. Appl Environ Microbiol 87:e03140-20. doi:10.1128/AEM.03140-20 33741621 PMC8208140

[25] Taniguchi Y , Choi PJ , Li G-W , Chen H , Babu M , Hearn J , Emili A , Xie XS . 2010. Quantifying E. coli proteome and transcriptome with single-molecule sensitivity in single cells. Science 329:533–538. doi:10.1126/science.1188308 20671182 PMC2922915

[26] Niesner R , Heintz A . 2000. Diffusion coefficients of aromatics in aqueous solution. J Chem Eng Data 45:1121–1124. doi:10.1021/je0000569

[27] Sangster J . 1989. Octanol-water partition coefficients of simple organic compounds. J Phys Chem Ref Data 18:1111–1229. doi:10.1063/1.555833

[28] Kube M , Heider J , Amann J , Hufnagel P , Kühner S , Beck A , Reinhardt R , Rabus R . 2004. Genes involved in the anaerobic degradation of toluene in a denitrifying bacterium, strain EbN1. Arch Microbiol 181:182–194. doi:10.1007/s00203-003-0627-3 14735297

[29] Rabus R , Kube M , Beck A , Widdel F , Reinhardt R . 2002. Genes involved in the anaerobic degradation of ethylbenzene in a denitrifying bacterium, strain EbN1. Arch Microbiol 178:506–516. doi:10.1007/s00203-002-0487-2 12420173

[30] Jumper J , Evans R , Pritzel A , Green T , Figurnov M , Ronneberger O , Tunyasuvunakool K , Bates R , Žídek A , Potapenko A , Bridgland A , Meyer C , Kohl SAA , Ballard AJ , Cowie A , Romera-Paredes B , Nikolov S , Jain R , Adler J , Back T , Petersen S , Reiman D , Clancy E , Zielinski M , Steinegger M , Pacholska M , Berghammer T , Bodenstein S , Silver D , Vinyals O , Senior AW , Kavukcuoglu K , Kohli P , Hassabis D . 2021. Highly accurate protein structure prediction with AlphaFold. Nature 596:583–589. doi:10.1038/s41586-021-03819-2 34265844 PMC8371605

[31] Koh S , Hwang J , Guchhait K , Lee E-G , Kim S-Y , Kim S , Lee S , Chung JM , Jung HS , Lee SJ , Ryu C-M , Lee S-G , Oh T-K , Kwon O , Kim MH . 2016. Molecular insights into toluene sensing in the TodS/TodT signal transduction system. J Biol Chem 291:8575–8590. doi:10.1074/jbc.M116.718841 26903514 PMC4861429

[32] Rabus R , Widdel F . 1995. Anaerobic degradation of ethylbenzene and other aromatic hydrocarbons by new denitrifying bacteria. Arch Microbiol 163:96–103. doi:10.1007/BF00381782 7710331

[33] Wöhlbrand L , Rabus R . 2009. Development of a genetic system for the denitrifying bacterium "Aromatoleum aromaticum" strain EbN1. J Mol Microbiol Biotechnol 17:41–52. doi:10.1159/000159194 18818489

[34] Schäfer A , Tauch A , Jäger W , Kalinowski J , Thierbach G , Pühler A . 1994. Small mobilizable multi-purpose cloning vectors derived from the Escherichia coli plasmids pK18 and pK19: selection of defined deletions in the chromosome of Corynebacterium glutamicum. Gene 145:69–73. doi:10.1016/0378-1119(94)90324-7 8045426

[35] Kovach ME , Elzer PH , Hill DS , Robertson GT , Farris MA , Roop RM , Peterson KM . 1995. Four new derivatives of the broad-host-range cloning vector pBBR1MCS, carrying different antibiotic-resistance cassettes. Gene 166:175–176. doi:10.1016/0378-1119(95)00584-1 8529885

[36] Oelmüller U , Krüger N , Steinbüchel A , Freidrich CG . 1990. Isolation of prokaryotic RNA and detection of specific mRNA with biotinylated probes. J Microbiol Methods 11:73–86. doi:10.1016/0167-7012(90)90050-G

[37] Ramakers C , Ruijter JM , Deprez RHL , Moorman AFM . 2003. Assumption-free analysis of quantitative real-time polymerase chain reaction (PCR) data. Neurosci Lett 339:62–66. doi:10.1016/s0304-3940(02)01423-4 12618301

[38] Berman HM , Westbrook J , Feng Z , Gilliland G , Bhat TN , Weissig H , Shindyalov IN , Bourne PE . 2000. The protein data bank. Nucleic Acids Res 28:235–242. doi:10.1093/nar/28.1.235 10592235 PMC102472

[39] Varadi M , Anyango S , Deshpande M , Nair S , Natassia C , Yordanova G , Yuan D , Stroe O , Wood G , Laydon A , Žídek A , Green T , Tunyasuvunakool K , Petersen S , Jumper J , Clancy E , Green R , Vora A , Lutfi M , Figurnov M , Cowie A , Hobbs N , Kohli P , Kleywegt G , Birney E , Hassabis D , Velankar S . 2022. AlphaFold protein structure database: massively expanding the structural coverage of protein-sequence space with high-accuracy models. Nucleic Acids Res 50:D439–D444. doi:10.1093/nar/gkab1061 34791371 PMC8728224

[40] Robert X , Gouet P . 2014. Deciphering key features in protein structures with the new ENDscript server. Nucleic Acids Res 42:W320–4. doi:10.1093/nar/gku316 24753421 PMC4086106

