# Supplementary material for: Sensitive and selective phenol sensing in denitrifying Aromatoleum aromaticum EbN1T
Source: Microbiol Spectr. 2023 Oct 12;11(6):e02100-23. doi: 10.1128/spectrum.02100-23 (PMC10715001; doi:10.1128/spectrum.02100-23)
Supplement: Fig. S1 — Anaerobic degradation pathways for phenol, p-cresol, and p-ethylphenol. [file spectrum.02100-23-s0001.pdf]

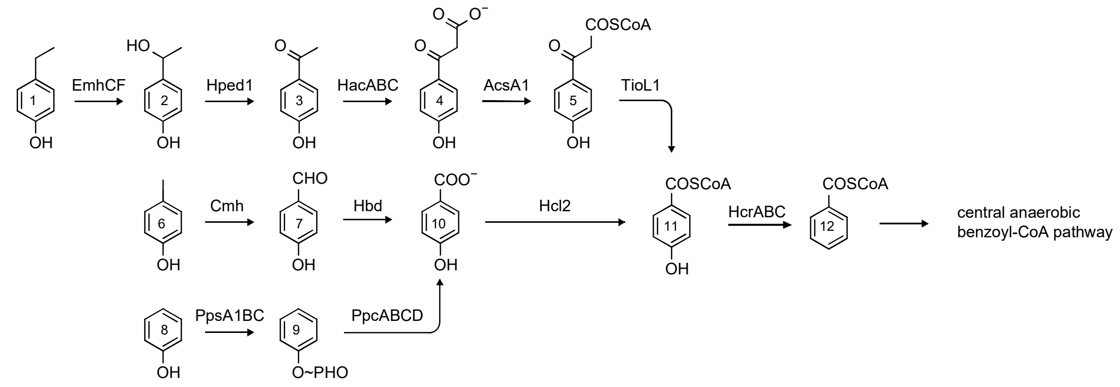

FIG S1 Anaerobic degradation pathways for phenol, *p*-cresol and *p*-ethylphenol in *A. aromaticum* EbN1^{T}. Compound names: 1, *p*-ethylphenol; 2, 1-(4-hydroxyphenyl)ethanol; 3, 4-hydroxyacetophenone; 4, 4-hydroxybenzoylacetate; 5, 4-hydroxybenzoylacetyl-CoA; 6, *p*-cresol; 7, 4-hydroxybenzaldehyde; 8, phenol; 9, phenylphosphate; 10, 4-hydroxybenzoate; 11, 4-hydroxybenzoyl-CoA; 12, benzoyl-CoA. Enzyme names are as described in legend to Fig. 1, except for HcrABC, 4-hydroxybenzoyl-CoA reductase.
