# Supplementary material for: Sensitive and selective phenol sensing in denitrifying Aromatoleum aromaticum EbN1T
Source: Microbiol Spectr. 2023 Oct 12;11(6):e02100-23. doi: 10.1128/spectrum.02100-23 (PMC10715001; doi:10.1128/spectrum.02100-23)
Supplement: Fig. S2 — Anaerobic cultivation of A. aromaticum EbN1T for targeted transcript analysis in response to phenol. [file spectrum.02100-23-s0002.pdf]

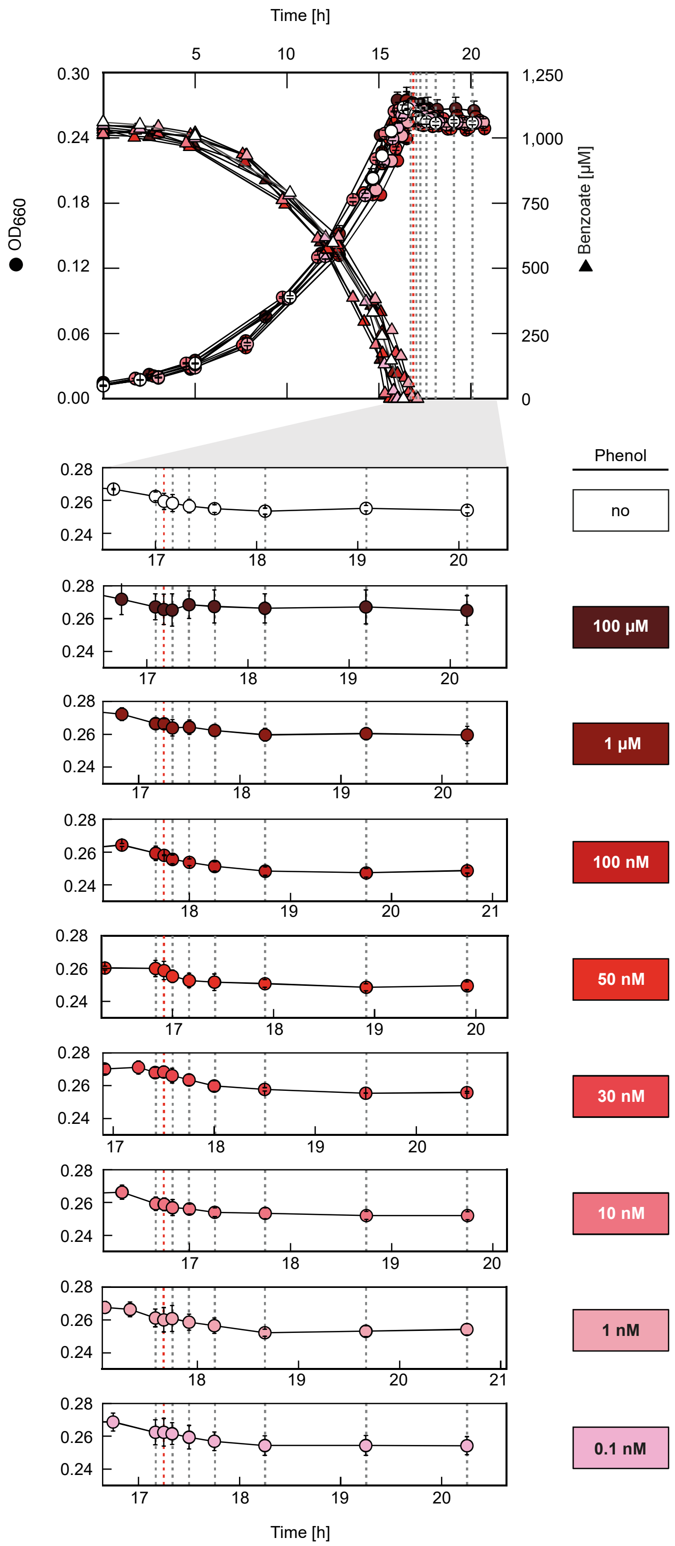

FIG S2 Anaerobic cultivation of *A. aromaticum* EbN1[T] for targeted transcript analysis in response to phenol. Growth was monitored by measuring OD_{660}. Upon depletion of benzoate after ~17.2 h, phenol was added (red dashed line) at a distinct concentration as indicated in the colored boxes. Grey dashed lines indicate the sampling time points for transcript analyses. For each tested condition, triplicate cultures were performed (note error bars, standard deviation). Benzoate depletion was determined via microHPLC.
