# Supplementary material for: Sensitive and selective phenol sensing in denitrifying Aromatoleum aromaticum EbN1T
Source: Microbiol Spectr. 2023 Oct 12;11(6):e02100-23. doi: 10.1128/spectrum.02100-23 (PMC10715001; doi:10.1128/spectrum.02100-23)
Supplement: Fig. S3 — Transcript profiles of the cmh gene in wild type and ΔpheR mutant of A. aromaticum EbN1T after a pulse with 100 μM phenol. [file spectrum.02100-23-s0003.pdf]

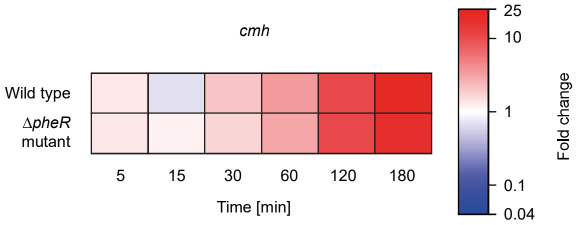

FIG S3 Transcript profiles of the *cmh* gene in wild type and Δ*pheR* mutant of *A. aromaticum* EbN1^{T} after a pulse with 100 µM phenol. Each data point is based on three biological replicates with three technical replicates each. Fold changes of transcript abundance are shown in Table S1.
