# Supplementary material for: Sensitive and selective phenol sensing in denitrifying Aromatoleum aromaticum EbN1T
Source: Microbiol Spectr. 2023 Oct 12;11(6):e02100-23. doi: 10.1128/spectrum.02100-23 (PMC10715001; doi:10.1128/spectrum.02100-23)
Supplement: Fig. S4 — No gratuitous induction of toluene- and ethylbenzene-catabolic genes with phenolic compounds in A. aromaticum EbN1T. [file spectrum.02100-23-s0004.pdf]

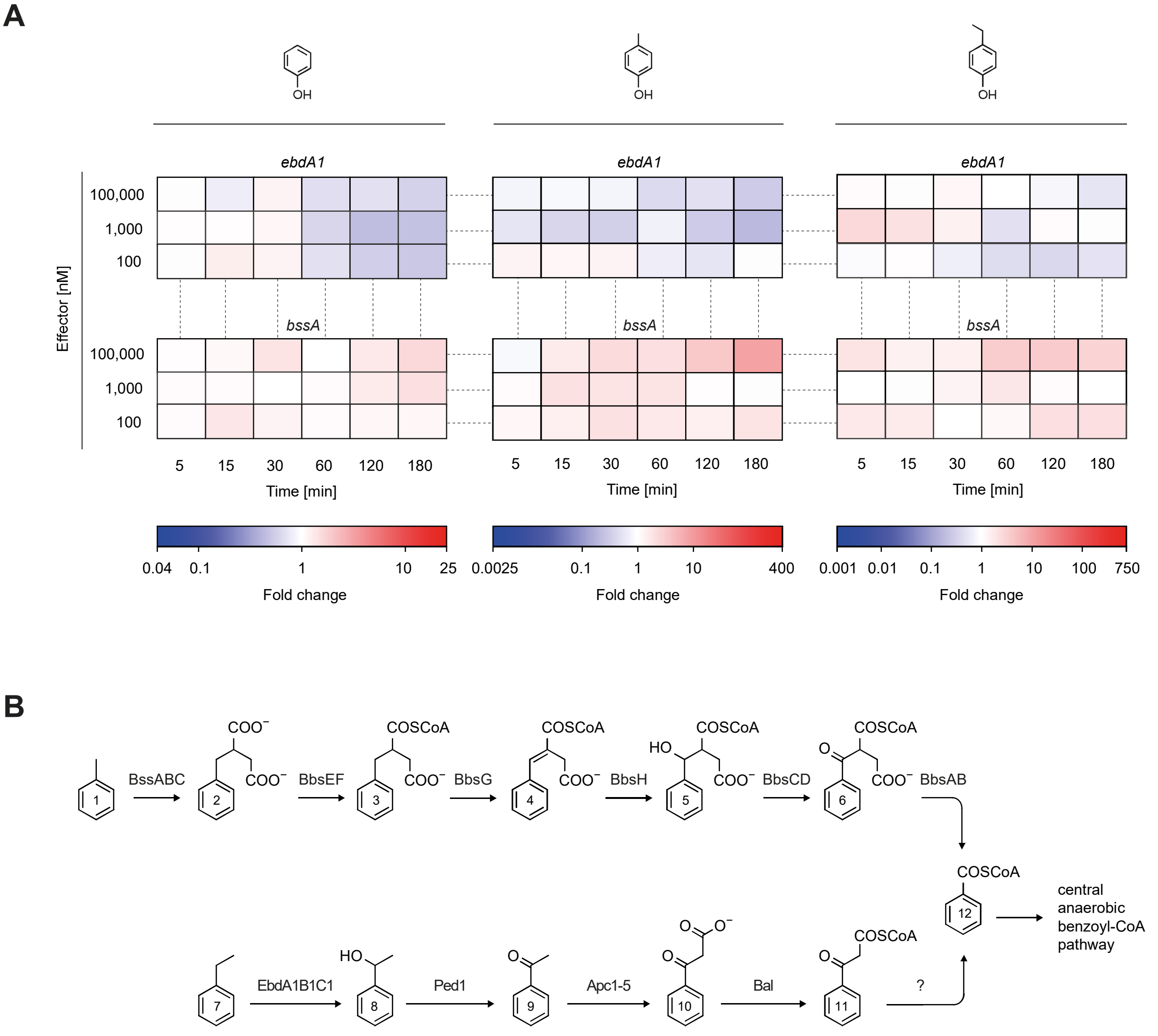

FIG S4 No gratuitous induction of 'toluene- and ethylbenzene-catabolic' genes with phenolic compounds in *A. aromaticum* EbN1^{T}. (A) Transcript profiles of the *ebdA1* and *bssA* genes in response to phenol, *p*-cresol and *p*-ethylphenol. Each data point is based on three biological replicates with three technical replicates each. The *ebdA1* and *bssA* genes encode the catalytic subunits of ethylbenzene dehydrogenase and benzylsuccinate synthase, respectively. Basic experimental settings were as described in legend to Fig. 4. (B) Anaerobic degradation pathway of toluene and ethylbenzene. Compound names: 1, toluene; 2, (*R*)-benzylsuccinate; 3, (*R*)-benzylsuccinyl-CoA; 4, (*E*)-phenylitaconyl-CoA; 5, 2-(α-hydroxyphenyl)methylsuccinyl-CoA; 6, (*S*)-2-benzoylsuccinyl-CoA; 7, ethylbenzene; 8, (*S*)-1-phenylethanol; 9, acetophenone; 10, benzoylacetate; 11, benzoylacetyl-CoA; 12, benzoyl-CoA. Enzyme names (toluene degradation): BssABC, benzylsuccinate synthase; BbsEF, (*R*)-benzylsuccinate CoA-transferase; BbsG, (*R*)-benzylsuccinyl-CoA dehydrogenase; BbsH, (*E*)-benzylidenesuccinyl-CoA hydratase; BbsCD, (*S*,*R*)-2-(α-hydroxybenzyl)succinyl-CoA dehydrogenase; BbsAB, (*S*)-2-benzoylsuccinyl-CoA thiolase. Enzyme names (ethylbenzene degradation): EbdA1B1C1, ethylbenzene dehydrogenase; Ped1, (*S*)-1-phenylethanol dehydrogenase; Apc1-5, acetophenone carboxylase; Bal, benzoylacetate-CoA ligase.
