# Supplementary material for: Sensitive and selective phenol sensing in denitrifying Aromatoleum aromaticum EbN1T
Source: Microbiol Spectr. 2023 Oct 12;11(6):e02100-23. doi: 10.1128/spectrum.02100-23 (PMC10715001; doi:10.1128/spectrum.02100-23)
Supplement: Fig. S5 — Rationale for incapacity of toluene and ethylbenzene sensory proteins to respond to phenolic compounds in A. aromaticum EbN1T. [file spectrum.02100-23-s0005.pdf]

A

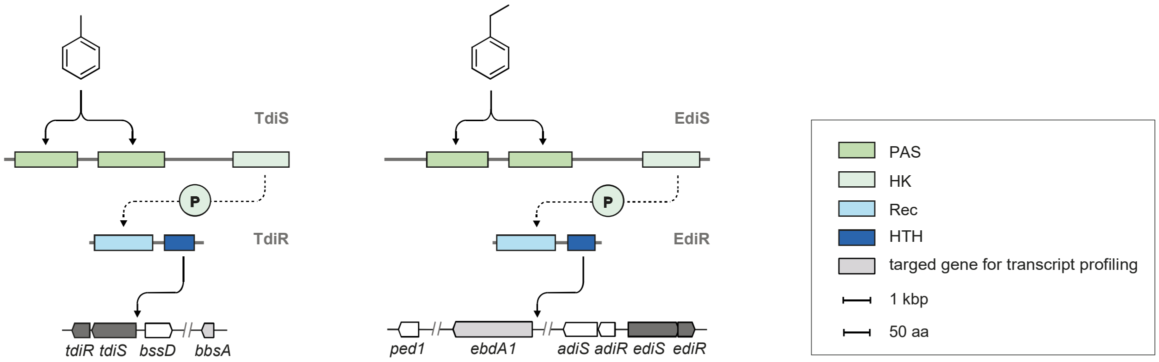

B

Regulator as query	Regulator	E value
MopR	PheR	0
MopR	PcrS	2e-28
MopR	EtpR	7e-150
MopR	EdiS	1.3
MopR	TdiS	5.7
PheR	EdiS	1.8
PheR	TdiS	4.5

C

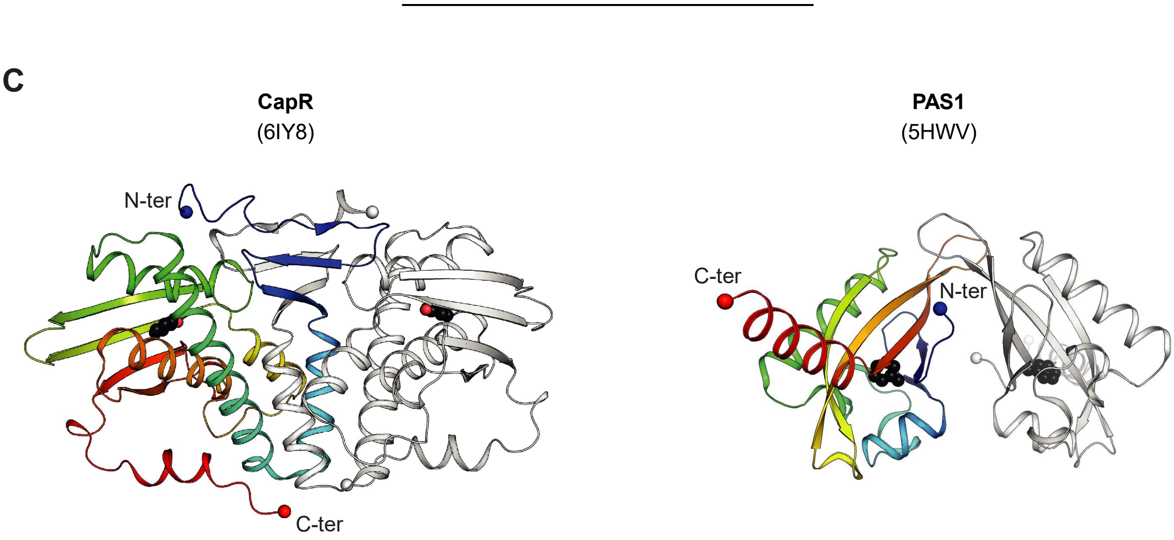

FIG S5 Rational for incapacity of toluene and ethylbenzene sensory proteins to respond to phenolic compounds in *A. aromaticum* EbN1^{T}. (A) Sensory/regulatory two-component systems controlling anaerobic degradation of toluene (TdiSR) and ethylbenzene (EdiSR). Functional domains of TdiSR and EdiSR are color coded and their coding genes marked in dark grey; explanation of functional domains is provided in legend to Fig. 1. 'Catabolic' genes selected for transcript profiling are highlighted in light grey. (B) Sequence comparison (BLAST) of phenol vs. alkylbenzene responsive regulators: MopR, PheR, PcrS and EtpR (all phenolic compounds), TdiS (toluene) and EdiS (ethylbenzene). (C) Overall folds of the sensory domains of phenol-binding CapR (PDB 6IY8) and toluene-binding PAS1 (PDB 5HWV) from *Pseudomonas putida*. Monomers of the dimeric proteins are grey vs. colored (gradient from blue N-terminus to red C-terminus). Large balls highlight phenol and toluene molecules modeled based on experimental data (black and red for carbon and oxygen atoms, respectively).
