# Supplementary material for: Sensitive and selective phenol sensing in denitrifying Aromatoleum aromaticum EbN1T
Source: Microbiol Spectr. 2023 Oct 12;11(6):e02100-23. doi: 10.1128/spectrum.02100-23 (PMC10715001; doi:10.1128/spectrum.02100-23)
Supplement: Fig. S6 — Shape and spatial expansion of phenol-binding pockets comparing models from crystal structures and AlphaFold predictions. [file spectrum.02100-23-s0006.pdf]

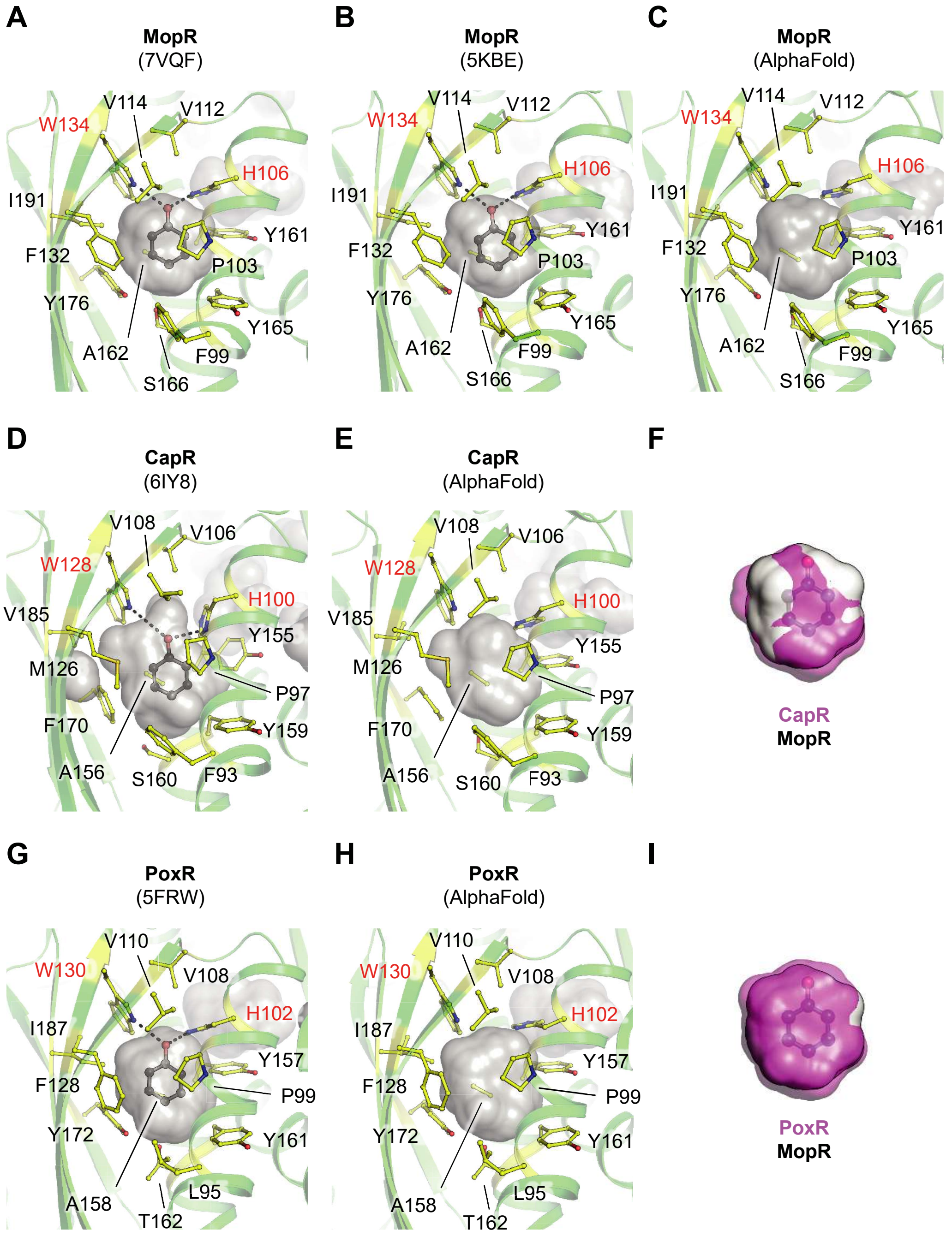

FIG S6 Shape and spatial expansion of phenol-binding pockets comparing models from crystal structures and AlphaFold predictions. (A) MopR (crystal structure, PDB 7VQF) from *Acinetobacter calcoaceticus*. (B) MopR (crystal structure, PDB 5KBE) from *A. calcoaceticus*. (C) MopR (AlphaFold model) from *A. calcoaceticus*. (D) CapR (crystal structure, PDB 6IY8) from *Pseudomonas putida*. (E) CapR (AlphaFold model) from *P. putida*. (F) Comparison of the cavity volume between MopR (experimental, solid grey surface) and CapR (AlphaFold model, transparent violet surface). (G) PoxR (crystal structure, PDB 5FRW) from *Cupriavidus necator*. (H) PoxR (AlphaFold model) from *C. necator*. (I) Comparison of the cavity volume between MopR (experimental, solid grey surface) and PoxR (AlphaFold model, transparent violet surface). The modeled ligand-accommodating cavity is indicated as a transparent grey surface, while the residues enclosing the cavity are highlighted in balls and sticks. The conserved His and Trp residues anchoring the hydroxy group of the ligand are labeled in red, and the dashes show the hydrogen bonds in the experimental models. Phenol, modeled based on experimental data (PDB 5KBE), with carbon and oxygen atoms colored in black and red, respectively.
