# Supplementary material for: Sensitive and selective phenol sensing in denitrifying Aromatoleum aromaticum EbN1T
Source: Microbiol Spectr. 2023 Oct 12;11(6):e02100-23. doi: 10.1128/spectrum.02100-23 (PMC10715001; doi:10.1128/spectrum.02100-23)
Supplement: Fig. S7 — Similarities of the overall sensory domain structures from different sensory/regulatory proteins for phenolic compounds comparing models from crystal structures and AlphaFold predictions. [file spectrum.02100-23-s0007.pdf]

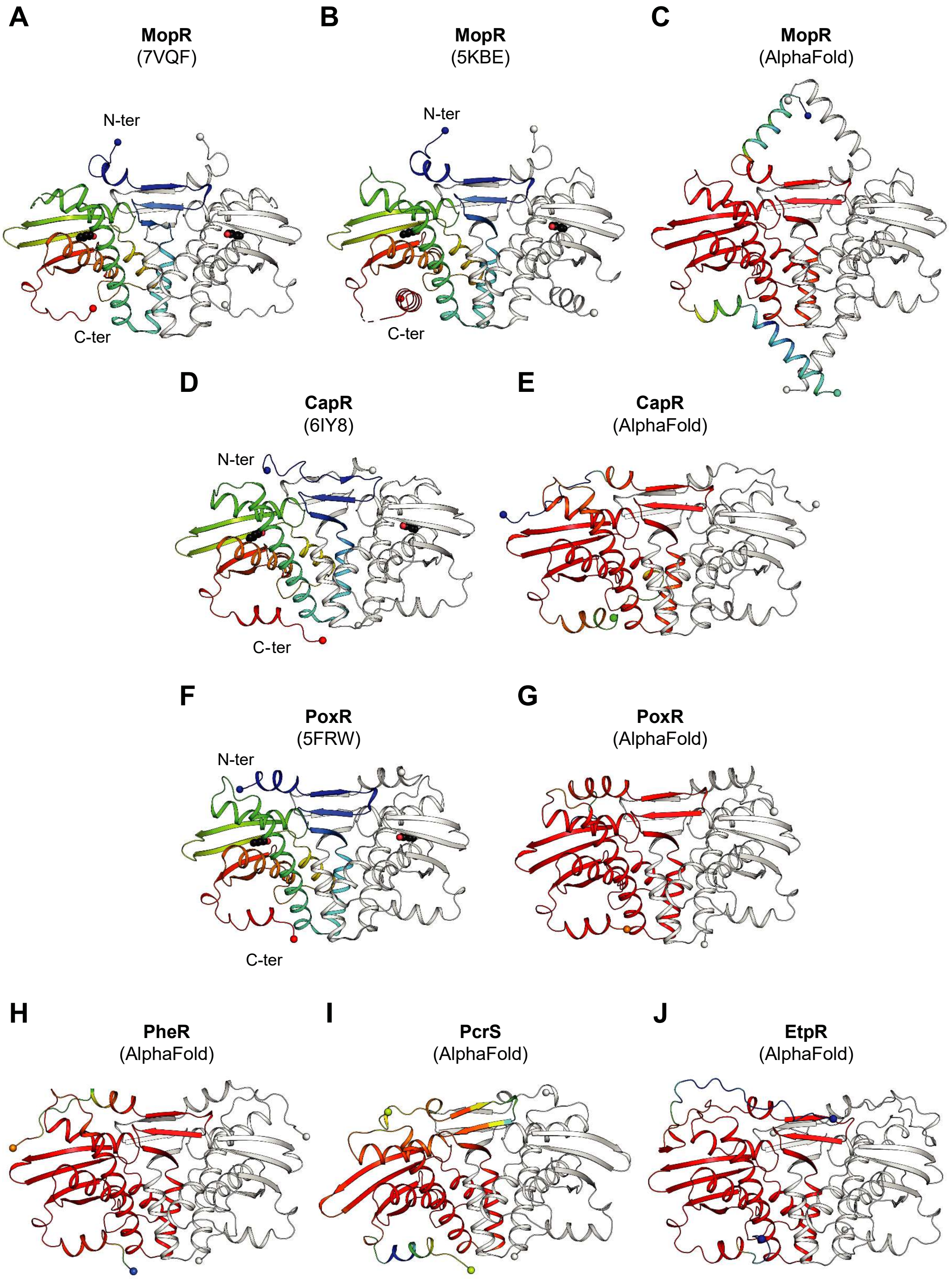

FIG S7 Similarities of the overall sensory domain structures from different sensory/regulatory proteins for phenolic compounds comparing models from crystal structures and AlphaFold predictions. MopR, CapR and PoxR are as described in the legend of Fig. S6; PheR, PcrS and EtpR are from *A. aromaticum* EbN1^{T}. All presented models were superposed first on the left monomer from MopR (7VQF, panel A). Large balls highlight phenol molecules modeled based on experimental data, and small balls point to the protein termini. Proteins are shown as homodimers in cartoons, with one monomer in white. In the experimental models, one monomer is colored from blue (N-terminus) to red (C-terminus). In the AlphaFold predicted models, one monomer is colored according to the confidence score of AlphaFold predictions from red (very high) to blue (low).
